# Listening to the community: Using formative research to strengthen maternity waiting homes in Zambia

**DOI:** 10.1371/journal.pone.0194535

**Published:** 2018-03-15

**Authors:** Nancy A. Scott, Taryn Vian, Jeanette L. Kaiser, Thandiwe Ngoma, Kaluba Mataka, Elizabeth G. Henry, Godfrey Biemba, Mary Nambao, Davidson H. Hamer

**Affiliations:** 1 Department of Global Health, Boston University School of Public Health, Boston, Massachusetts, United States of America; 2 Zambia Center for Applied Health Research and Development (ZCAHRD), Lusaka, Zambia; 3 Department of Public Health, Ministry of Health, Lusaka, Zambia; 4 Department of Medicine, Boston Medical Center, Boston, Massachusetts, United States of America; King's College London, UNITED KINGDOM

## Abstract

**Background:**

The WHO recommends maternity waiting homes (MWH) as one intervention to improve maternal and newborn health. However, persistent structural, cultural and financial barriers in their design and implementation have resulted in mixed success in both their uptake and utilization. Guidance is needed on how to design a MWH intervention that is acceptable and sustainable. Using formative research and guided by a sustainability framework for health programs, we systematically collected data from key stakeholders and potential users in order to design a MWH intervention in Zambia that could overcome multi-dimensional barriers to accessing facility delivery, be acceptable to the community and be financially and operationally sustainable.

**Methods and findings:**

We used a concurrent triangulation study design and mixed methods. We used free listing to gather input from a total of 167 randomly sampled women who were pregnant or had a child under the age of two (n = 59), men with a child under the age of two (n = 53), and community elders (n = 55) living in the catchment areas of four rural health facilities in Zambia. We conducted 17 focus group discussions (n = 135) among a purposive sample of pregnant women (n = 33), mothers-in-law (n = 32), traditional birth attendants or community maternal health promoters (n = 38), and men with a child under two (n = 32). We administered 38 semi-structured interviews with key informants who were identified by free list respondents as having a stake in the condition and use of MWHs. Lastly, we projected fixed and variable recurrent costs for operating a MWH.

Respondents most frequently mentioned distance, roads, transport, and the quality of MWHs and health facilities as the major problems facing pregnant women in their communities. They also cited inadequate advanced planning for delivery and the lack of access to delivery supplies and baby clothes as other problems. Respondents identified the main problems of MWHs specifically as over-crowding, poor infrastructure, lack of amenities, safety concerns, and cultural issues. To support operational sustainability, community members were willing to participate on oversight committees and contribute labor. The annual fixed recurrent cost per 10-bed MWH was estimated as USD543, though providing food and charcoal added another $3,000USD. Respondents identified water pumps, an agriculture shop, a shop for baby clothes and general goods, and grinding mills as needs in their communities that could potentially be linked with an MWH for financial sustainability.

**Conclusions:**

Findings informed the development of an intervention model for renovating existing MWH or constructing new MWH that meets community standards of safety, comfort and services offered and is aligned with government policies related to facility construction, ownership, and access to health services. The basic strategies of the new MWH model include improving community acceptability, strengthening governance and accountability, and building upon existing efforts to foster financial and operational sustainability. The proposed model addresses the problems cited by our respondents and challenges to MWHs identified by in previous studies and elicits opportunities for social enterprises that could serve the dual purpose of meeting a community need and generating revenue for the MWH.

## Introduction

Governments, funders and implementers of global health programs are striving to incorporate long-term sustainability strategies in program development and implementation efforts [1]. Likewise, as evidence-based interventions are implemented in new and different contexts, researchers are adapting traditional methods of inquiry to identify the elements of sustainability of public health programs [1–3]. Obtaining community input and engaging stakeholders are demonstrated methods that can improve implementer understanding of a problem to be addressed and increase community ownership of a program, particularly those designed to address behaviors and practices influenced by cultural norms [3–6]. Yet methods to adequately plan for sustainability are not often employed due to financial or time constraints. Moreover, there is little evidence to guide implementers in how to employ these methods. This article documents how formative research was used to design a community-based intervention in rural Zambia to address barriers to accessing skilled delivery care, and to inform the creation of systems to increase the overall operational and financial sustainability of the intervention.

While the World Health Organization (WHO) recommends ‘skilled care at every birth’ [7], pregnant women in Zambia face obstacles to seeking and accessing skilled delivery care, including reaching a health facility that is adequately staffed. Although 50% of the rural population in Zambia lives within 5 kilometers (km) of a health facility, the average distance to a health facility that is equipped for safe deliveries and offers emergency obstetric and neonatal care (EmONC) is more than 15 km [8]. The odds of a woman delivering in a facility in rural Zambia decreases as distance to the facility increases [9]. Just two-thirds of women deliver at a health facility and 64% deliver with a skilled attendant trained in managing complications [10].

Social and cultural factors, accessibility of facilities including distance and cost, and quality of health facilities all interact to influence a woman’s decision to seek care, access care, and receive appropriate and adequate treatment, as articulated in the Three Delays conceptual model [11–17]. In Zambia, specifically, distance and poor road conditions [8,18]; transportation availability and cost [16,17]; indirect costs of delivering at a facility, such as the need for baby clothes [16]; women’s autonomy to make decisions about their maternal care [19]; and the low perceived quality of healthcare services [16,18] are documented barriers to women seeking and accessing care. Designing and implementing effective programs to access skilled delivery care is challenging and requires an approach that will address the multi-dimensional barriers, be acceptable to the community, and have the potential to be sustainable. Stakeholders that play a critical role in this intervention include Ministry of Health officials; health facility staff; community health workers (CHWs); Safe Motherhood Action Groups (SMAGs), a cadre of non-clinical volunteers that work at the community level specifically on outreach for maternal health issues; traditional, church, civic and political leaders; and community members themselves.

The WHO has recommended maternity waiting homes (MWH) as one intervention to improve maternal and newborn health. MHWs are residential accommodations adjacent to a health facility capable of managing labor and delivery complications and neonatal complications. In the event of labor complications, a woman at a MWH is closer to skilled care for proper management or, when needed, referral to higher level care than she would have been at home. MWHs have been implemented throughout Africa, Latin America, and Asia [20–22]. However, persistent structural, cultural and financial barriers in their design and implementation have resulted in mixed successes in MWH utilization by pregnant women [23–25].

MWH interventions that do not meet community standards of acceptability are unlikely to be successful [23,26]. With the recent WHO recommendation of MWHs [27], guidance is needed on how to systematically design a MWH intervention that is acceptable to community standards and sustainable for long-term use [25,26].

Designing sustainable, locally acceptable and culturally appropriate public health programs requires rigorous formative research. Guided by the conceptual framework for sustainability of public health programs (S1 Fig), outlined by Scheirer and Dearing, we sought stakeholder input on factors that affect program sustainability [1]. This framework details that in addition to financial resources and inputs such as the evidence base of the intervention, capacity of the organization and prior relationships, other factors such as the intervention characteristics, organizational support and environmental support interact within a larger environment to affect sustainability outcomes [1]. While not exhaustive of all known and unknown factors influencing sustainability, this framework provided a roadmap of major constructs for us to examine for our design of a sustainable intervention. Through formative research, we aimed to design a MWH intervention that could 1) overcome barriers to access to facility delivery; 2) be acceptable to the community; and 3) be both financially and operationally sustainable.

## Methods

### Setting

Our study was conducted in the contiguous districts of Choma and Kalomo in Southern Province, Zambia. At the time of data collection, Choma District included what is now the administrative district of Pemba, and Kalomo District included what is now the administrative district of Zimba. Choma and Kalomo Districts are primarily rural with some peri-urban areas. Choma District has a population of 247,860 [28], with approximately 11,830 births per year [29]. Choma District has 33 rural health centers (RHC), three hospitals, and eight health posts [30]. Kalomo District has a population of 258,570 [28], with approximately 13,737 births per year [29]. Kalomo District has 31 RHC, two hospitals, and three health posts (30). Deliveries typically happen at the rural health centers, with referrals to hospitals when necessary. Twenty-eight of the health facilities in Choma and Kalomo Districts had an existing MWH structure, while three had no physical structure but the health facility staff allowed pregnant women to sleep in the wards at night [31]. In general, the existing MWHs had poor infrastructure with few amenities such as beds or mosquito nets [31].

### Study design and data collection

Over a period of 5 months in 2013–2014, we employed mixed methods, using a concurrent triangulation design wherein methods were applied at the same time to confirm and cross-validate the findings [32]. The approach included gathering community input, engaging key stakeholders, and creating cost projections for operating a MWH. The sample size for each qualitative data collection method was estimated to ensure we reached saturation or predictability, the point at which no new themes or issues emerge [33].

### Community input

In the catchment areas of four randomly selected facilities with existing MWHs, we approached every nth household (dependent on catchment size) to gather community input from a total of 167 randomly sampled women who were pregnant or had a child under the age of two, men with a child under the age of two, and community elders all from three distances from the health facilities: within 5 km, between 5 and 10 km, and greater than 10 km. We initially estimated a sample of 120–200 respondents, and reached saturation after 167. After administering a short survey, we used free listing (FL) [34] (S1 File), in which each respondent generated an exhaustive list of responses to the following broad, open-ended questions: 1) What are the biggest problems for pregnant women through delivery in your community?; 2) What do people in your community know or believe about MWHs at health facilities?; and 3) What businesses or services are needed but not currently available in your community? This third question was used to explore possible revenue generating activities that could help support the MWH financially. FL results were used to inform the development of the focus group discussion (FGD) and key informant interview (KII) guides, and generate a sample of key informants (S2 File and S3 File).

In the same catchment areas, we also conducted 17 FGDs among a purposive sample of pregnant women, mothers-in-law, traditional birth attendants (TBAs) or community members who were part of a cadre of trained maternal health promoters called Safe Motherhood Action Groups (SMAGs) [35], and men with children under the age of two. FGDs captured information on perceptions of place of delivery, barriers and facilitators to access, and perceptions of MWHs.

### Stakeholder engagement

We conducted 38 semi-structured interviews with key informants who were recommended most frequently by the FL respondents, and who were considered to have a stake in improvement in the condition and use of MWH in their communities. These key informants included health facility staff, headmen, and TBAs, among others. KIIs were used to more deeply explore the ideas that emerged from the FL.

### Cost projections

We collected cost information from government and private sector records to estimate the fixed and variable recurrent costs of operating a basic, functional MWH, and to inform a cost recovery plan (S4 File). To inform a MWH financial sustainability strategy, we captured willingness to pay among all respondents; detailed results are presented elsewhere [36]. We anticipated that the communities might be able to operate a small business or income generating activity (IGA), using the profits to contribute to the cost of operating the MWH.

The cost-volume-profit analysis included only the recurrent costs per month, assuming that any MWH major renovations or construction would be funded entirely by an outside source. We included the cost of the specific components detailed by respondents as necessary for good quality (e.g., bed linens, locks, etc.).

### Data collection methods

Nine local data collectors fluent in English and Tonga, the local language, attended a 5-day training in research ethics, research methods, and quantitative and qualitative interviewing techniques immediately before data collection. FL questions were designed to elicit perspectives on problems that pregnant women face in the community, community perceptions of MWHs, and ideas for sustainability. FGD and KII guides were initially developed based on a review of the maternal health literature, then refined based on themes that emerged during the analysis of FL results. Socio-demographic characteristics of all respondents were measured. Costs were collected from expenditure records or direct quotations from vendors.

### Data management and analysis

Socio-demographic data were captured in Microsoft® Excel and analyzed in SAS v9.1.3 [37]. FL responses were captured on paper and analyzed nightly using pile sorting wherein responses from each participant were written on individual cards, shuffled, and grouped by data collectors into piles of similar constructs to detect emerging themes [34,38] (S1 Dataset). KII and FGD transcripts were coded for themes and the themes were linked into a theoretical model guided by the Three Delay model and the sustainability framework [1,39]. Qualitative data were translated and transcribed into Microsoft® Word and coded and analyzed in NVivo v10 [40].

We performed a cost-volume-profit analysis to determine the fixed and variable costs and revenue needed to function at various levels of activity [41]. We then conducted break-even analyses based on alternative assumptions and potential revenue stream identified by the respondents. Cost data were captured in Microsoft® Excel.

### Ethical considerations

Ethical approval was obtained from the Boston University Institutional Review Board (IRB) and the ERES Converge IRB in Zambia. Prior to data collection, we secured letters of support from the Ministry of Health at the national, provincial and district levels. We also had support and approval from the four Chiefs (traditional leaders) overseeing the catchment areas where we had planned research activities. We obtained verbal informed consent for each participant.

## Results

### Study sample

We had a total of 167 FL respondents and 135 FGD respondents (Table 1). The median age of women respondents was slightly younger than men in both FLs and FGDs. Among FGD respondents, the median age of TBA/SMAG participants and mother-in-law participants was higher than women and men with children under two years.

**Table 1:** Characteristics of the free list and focus group discussion respondents.

	Free List Participants			Focus Group Discussion Participants			
	Women (n = 59)	Men (n = 53)	Elders (n = 55)	Women (n = 33)	Men (n = 32)	TBA/SMAG (n = 38)	Mothers-in -law (n = 32)
Age, median (IQR)	25 (22,33)	32 (28,37)	63 (56,70)	23 (18,29)	34 (42,44)	50 (45,56)	57 (51,59)
Male, n (%)	-	53 (100)	29 (53)	-	32 (100.0)	7 (18.4)	-
Marital status:							
*Married*, *n (%)*	54 (91.5)	53 (100.0)	41 (74.6)	30 (90.1)	32 (100.0)	29 (76.3)	18 (56.3)
*Widowed or divorced*, *n (%)*	3 (5.1)	0 (n/a)	14 (25.5)	1 (3.0)	0 (n/a)	9 (23.7)	12 (37.5)
*Single*, *n (%)*	2 (3.4)	0 (n/a)	0 (n/a)	2 (6.1)	0 (n/a)	0 (n/a)	1 (3.1)
Distance from health facility in kilometers, median (range)	8.6 (1, 22)	7.7 (1, 20)	7.2 (1, 23)	-	-	-	-

Sex and job title were the only descriptive variables collected from the KIIs (n = 38). Of the KIIs 63% were male; 16 were health facility staff, 9 CHWs, 4 traditional leaders, 5 other types of community leaders, and 4 other respected community members.

### Problems for pregnant women in the communities

In response to the FL question about problems that pregnant women face, nearly half of all respondents cited distance and poor roads from home to the health facility (49.7%) and no ambulance (61.1%) as problems (Table 2). The 10 most frequently identified problems were fairly consistent across respondent groups, although men cited lack of ambulance transportation more commonly than women (84.9% compared to 47.5%). Other frequently cited problems included poor quality MWHs and health facilities.

**Table 2:** Ten most frequently mentioned problems for pregnant women by type of FL respondent, n(%).

FL question 1: What are the biggest problems for pregnant women through delivery in your community?		Women (n = 59		Men (n = 53)		Elders (n = 55)		Total (n = 167)	
1	No ambulance to get pregnant woman from home to RHC* or RHC* to hospital/no transport to book	28	(47.5)	45	(84.9)	29	(52.7)	102	(61.1)
2	Long distance from home to clinic; Walking by foot is far; poor roads and bridges	29	(49.2)	28	(52.8)	26	(47.3)	83	(49.7)
3	The maternity home is small—the women do not fit	22	(37.3)	20	(37.7)	17	(30.9)	59	(35.3)
4	No money to buy baby clothes, CDK^+^ or other supplies required at clinic^++^	23	(39.0)	17	(32.1)	7	(12.7)	47	(28.1)
5	There are few health staff at the health facility	19	(32.2)	17	(32.1)	8	(14.5)	44	(26.3)
6	There are no beds in the maternity home; women sleep in the floor	16	(27.1)	13	(24.5)	13	(23.6)	42	(25.1)
7	Maternity home kitchen is too small	14	(23.7)	6	(11.3)	12	(21.8)	32	(19.2)
8	Small capacity of maternity ward	12	(20.3)	8	(15.1)	4	(07.3)	24	(14.4)
9	Delay in being attended to by health staff at clinic	6	(10.2)	9	(17.0)	8	(14.5)	23	(13.8)
10	Poor clinic infrastructure: no water, no electricity, no cell network	10	(16.9)	5	(9.4)	7	(12.7)	22	(13.2)

* RHC = rural health center

^+^ CDK = clean delivery kit

^++^ Other items reported by FL to be required include razor blade, cotton wool, fabric for cleaning, bucket, disinfectant

FGD and KII respondents also perceived long distances, impassable roads, limited transport or money for transport as problems for pregnant women in their communities, as illustrated by the quotes below:

“If you are in labor, it is difficult to walk long distances on foot. You can start off on time but because of the long distance you can end up delivering on the way.”–*KII*, *CHW*, *female*.

“Some [women], they have no support from the family…I remember there was one woman who was told from [antenatal care] that you have to deliver at the hospital, but the husband refused to give her money. He said, ‘I don’t have the money,’ till when she went into labor and she was brought here to the clinic. The nurses said, ‘We referred you to [the] hospital,’ but she died as they were still trying to look for transport money.”–*FGD*, *community elder*, *female*

Other key informants said that transport (ox carts, motorbikes, cars) is available in the communities, but not typically arranged in advance, and therefore perceived as unavailable. KIIs with chiefs and headmen raised the issue of limited advance planning for delivery and suggested the solution was not providing transport or vouchers, but rather strengthening individual and community planning efforts.

Of FL respondents, 28% expressed that lack of money to purchase baby clothes or other supplies was a problem for pregnant women in the communities. KII and FGD respondents corroborated this finding by explaining that supplies at the health facility were often inconsistently available, and that nurses expected a pregnant woman to bring baby clothes for her newborn. Therefore, women are expected, though not required, to bring their own delivery supplies and baby clothes.

“The clinic runs out of surgical gloves, cord clamps, etc., and women are requested to bring them at time of delivery. They should also bring a plastic basin and bucket, soap, Jik [a disinfectant], etc. So, if a woman cannot afford these requirements, they will deliver at home.”–*KII*, *midwife*, *female*

FGD respondents suggested it was challenging to procure the items and they feel embarrassed if they are unable to obtain the items required for delivery as illustrated below.

“Yes, it is costly for some of us because we cannot manage to buy baby clothes. So we are shy to send our wives to the clinic without those new clothes; instead we tell our wives to deliver at home.”–*FGD*, *male with child under the age of 2*

“Some they don’t have baby clothes because their husbands don’t buy. Now if I go and stay at the shelter when I deliver, the nurse will ask for baby clothes what am I going to say?”–*FGD*, *community elder*, *female*

Several FL responses relate to negative perceptions about the clinic in general, including infrastructure, space and inadequate staffing:

“The health workers are very few to offer quality services to pregnant mothers because the same nurse will be required to go to OPD [outpatient department], antenatal, deliveries, HART [highly active antiretroviral therapy] patients, and meetings.”–*KII*, *CHW*, *male*

“Yes it’s true the staff are few. Sometimes the nurse is very tired and can go to rest at home. As a result, the person who escorts the mother ends up conducting the delivery.”—*KII*, *CHW*, *female*

### Community perceptions of MWHs

In response to the FL question about general perceptions of MWHs, there was a mix of positive and negative responses. Nearly 44% of all respondents thought MWHs were helpful or good, but the most frequently mentioned responses were that they were small and not used exclusively for pregnant women (Table 3).

**Table 3:** Top 10 most frequently mentioned beliefs about MWHs as indicated by women, men, and elder Free List respondents, n (%).

	FL question 2: What do people in your community know or believe about maternity waiting homes?	Women (n = 59)		Men (n = 53)		Elders (n = 55)		Total (N = 167)	
1	MWH is small	19	(32.2)	30	(56.6)	30	(54.5)	79	(47.3)
2	MWH is for the community, anyone can stay there not just pregnant women	24	(40.7)	19	(35.8)	34	(61.8)	77	(46.1)
3	MWH is helpful and good	31	(52.5)	21	(39.6)	21	(38.2)	73	(43.7)
4	No beds at the MWH, women sleep on the floor	20	(33.9)	13	(24.5)	15	(27.3)	48	(28.7)
5	MWH is for pregnant women and those taking care of the sick	13	(22.0)	14	(26.4)	0	(0.0)	27	(16.2)
6	MWH is community property / built by the community	5	(8.5)	16	(30.2)	1	(1.8)	22	(13.2)
7	No kitchen at the MWH/kitchen is small	4	(6.8)	6	(11.3)	12	(21.8)	22	(13.2)
8	MWH has a spell put on it to make women who stay there go off labor (have contractions start then stop)	10	(16.9)	8	(15.1)	2	(3.6)	20	(12.0)
9	No power (electricity) at the MWH	6	(10.2)	7	(13.2)	6	(10.9)	19	(11.4)
10	MWH is haunted	5	(8.5)	5	(9.4)	8	(10.8)	14	(8.4)

Generally, FL responses with negative connotations fell into themes of comfort, safety, and cultural appropriateness. Comfort, particularly around overcrowding, was the primary theme elicited in the FL responses and corroborated by FGD and KII respondents. They explained that the MWHs were crowded, had no beds or mattresses, limited access to water, and were generally uncomfortable:

“In my opinion, some women, when they look at how the [MWH] is crowded and the space is so small, they decide to stay home. By the time they think of coming to the clinic [it is too late, and] they deliver at home.”–*FGD*, *SMAG member*, *female*

“[MWHs have] no proper place to sleep; [women] just sleep like prisoners.”–*FGD*, *TBA*, *male*

“The reason why some women don’t use the shelter is because there are no beds, no power for lighting, and limited beddings [linens and blankets]. The shelter is small.”–*KII*, *TBA*, *female*

**“**Yes, there are a lot of problems at the shelter. We sleep on the floor: there are no beds. In the morning, our bodies are sore. That can prevent mothers from coming and staying at the shelter.”–*FGD*, *woman with a child under the age of 2*

KII and FGD respondents identified the need for basic amenities such as beds, toilets, and lighting, as well as operational needs such as a management process for routine operations and maintenance. Additionally, KII and FGD respondents frequently suggested the provision of food, charcoal and a space for women to cook. FGD and KII respondents also perceived that safety at the MWHs was a concern. Most had limited lighting, there was no lockable space for women to keep their belongings, travelers sometimes shared the same space, and there were no lockable doors or windows.

KII and FGD suggestions to improve safety included the provision of electricity or sufficient lighting, mosquito nets, lockable doors and windows, and lockable cupboards to prevent theft of personal items.

“They steal from each other. The community [should] contribute and buy lockable lockers.”–*KII*, *Traditional Leader*, *male*

Additionally, FGD and KII respondents elaborated on cultural issues raised by the FL respondents. First, respondents believed it was culturally inappropriate to house pregnant women with patient families, travelers or even recently delivered women. Second, over 15% of women and men listed that staying at a MWH would delay delivery. This perception might arise from issues around estimating delivery dates. KII respondents perceived that health facility staff members have difficulties estimating the delivery date, largely because women misestimate their last menstrual period. Other key informants thought that health facility staff deliberately give the wrong delivery date in an effort to ensure that women show up for delivery with enough time. With earlier presentation, women stay at the shelter longer than expected, resulting in a belief that the shelter itself is “cursed” to cause delayed delivery.

“Some women fear to come and stay at the shelter, saying some old women who come there use charms to delay other women’s delivery.”–*KII*, *clinic clerk*, *male*

To improve cultural appropriateness, KII and FGD respondents suggested having MWHs for pregnant women only and separate shelters for other patients:

“We need a big shelter specifically for pregnant women so that we don’t mix with those who have come to look after the sick”–*FGD*, *woman with a child under the age of 2*

### Operational sustainability of MWH

Respondents suggested several ways in which to manage operations. Almost all options involved the health facility staff and headmen:

“There should be a small committee of maybe 5 people, but the facility staff [should] be involved and should lead. One staff to join the committee, if community members leading it have problems because of the limited knowledge. But people should understand this shelter is ours. They should not remove anything from there.”—*FGD*, *man with a child under the age of 2*

“Women, families and other community members can contribute to the maintenance of the shelter by working together where need arises [and] by cleaning the surroundings.”—*FGD*, *man with a child under the age of 2*

“To work together with the community, the facility staff will see if there is something that needs to be done at the shelter, like cleaning. They will inform the headman, then the headman will tell their subjects that.”—*FGD*, *woman with a child under the age of 2*

### Financial sustainability of MWH

The annual fixed recurrent cost per 10-bed shelter was estimated as $543, excluding a stipend for a MWH coordinator or on-site staff (Table 4). Assuming 25 users per month and an average length of stay of 10 days, estimates derived from key informants and health facility staff, projected annual variable costs would increase by $1,500 if food were provided, double that ($3,000) if both food and charcoal for cooking were provided.

**Table 4:** Estimated fixed and variable costs of operating a MWH.

Additional Fixed Costs per Facility	Estimated purchasing price ($USD) per year
Mosquito nets	100
Bed linens	200
Braziers-for cooking	17
Blankets	100
Locks for cupboards	50
Pots/pans	17
Cleaning supplies	60
**TOTAL RECURRING COSTS per Year**	**$543**
**TOTAL RECURRING COSTS per Month**	**$45**
**Variable Cost per User**	**Estimated purchasing price ($USD) per User**
Food	$5.00
Charcoal for cooking	$5.00
**TOTAL VARIABLE COSTS per User**	**$20.00**

KIIs and FGD respondents suggested recovering costs through government, community, and individual contributions [37], and IGAs. The most cited businesses/services mentioned, which we deemed might generate income to support a MWH, included: 1) a shop selling agricultural goods; 2) a shop selling general goods, including delivery supplies and baby clothes; 3) a mill for grinding maize; and 4) a market for selling produce (Table 5).

**Table 5:** Most frequently cited business or services needs as indicated by women, men, and elder Free List respondents, n (%).

FL Question: What businesses or services are needed but not currently available in your community?		Women (n = 59)		Men (n = 53)		Elders (n = 55)		Total (n = 167)	
1	Functioning water pump	30	(50.8)	19	(35.8)	31	(56.4)	80	(47.9)
2	Shop selling seeds and fertilizer	17	(28.8)	23	(43.4)	19	(34.5)	59	(35.3)
3	Health post	18	(30.5)	10	(18.9)	19	(34.5)	47	(28.1)
4	Shops to buy CDK, baby clothes, and general goods	19	(32.2)	10	(18.9)	17	(30.9)	46	(27.5)
5	Dams to preserve water for livestock and gardening	5	(08.5)	9	(17.0)	10	(18.2)	24	(14.4)
7	An ambulance	7	(11.9)	7	(13.2)	8	(14.5)	22	(13.2)
6	Mill for grinding maize	7	(11.9)	10	(18.9)	4	(07.3)	21	(12.6)
8	A bridge	3	(05.1)	8	(15.1)	8	(14.5)	19	(11.4)
9	Market for selling maize & produce	3	(05.1)	4	(7.5)	11	(20.0)	18	(10.8)
10	A preschool for small children	8	(13.6)	6	(11.3)	2	(03.6)	16	(9.9)

## Discussion

This study used formative research to systematically gather stakeholder input on community needs, barriers and facilitators in order to incorporate a plan to improve community ownership and sustainability in the design of a MWH intervention in Zambia. Stakeholders frequently mentioned problems of distance, poor roads, transport, and poor quality MWH and health facilities as problems facing pregnant women in their seeking care, supporting the findings from previous studies in Zambia or similar contexts [8,16–18,42]. Inadequate advanced planning for delivery, and lack of access to delivery supplies and baby clothes were also problems. Though new baby clothes are not formally required by health facilities, there is a clear perception among respondents that new baby clothes and multiple cloths to dry and wrap the newborn are required or not having them would be, in some way, stigmatizing. Community perceptions of MWH were mixed with the main problems being over-crowding, lack of adequate infrastructure and amenities, safety concerns, and cultural issues (e.g., mixing pregnant women with other hospital visitors needing a place to stay). To support operational sustainability, community members expressed a willingness to participate on oversight committees and contribute labor. To begin exploring financial sustainability, we identified the recurrent costs of running a MWH and identified possible community-led IGAs which might support the MWH costs, including an agricultural/general goods store and maize mill.

Based on our findings, we developed a preliminary MWH model, management structure, and financing strategy. We then held participatory meetings with 23 select stakeholders, some of whom had participated in the data collection activities, and additional government representatives to solicit input into refining the intervention design [34].

The resulting MWH model has specific actions to respond to community input, improve governance and accountability, and build on existing structures to maximize ownership and sustainability (Table 6). The model is a non-medical intervention for the renovation of an existing MWH or the construction of a new MWH that meets community standards of safety, comfort and services offered. We also sought to ensure that the model was aligned with current Zambian government policies related to facility construction, ownership, and access to health services. We deliberately chose not to include the provision of food in the model because of the potential implications on financial sustainability, though we recognize this could be a potential challenge to utilization.

**Table 6:** MWH intervention model derived from formative results.

Strategy	Objective	Specific Actions
**1. Respond to community input to improve acceptability**	Make shelters safer	• Include lockable cupboards, doors, windows, a fence and lighting • Implement a routine maintenance and repair plan (including connection to district budget, other ways to assure maintenance)
	Make shelters comfortable	• Provide mattresses, mosquito nets, cooking utensils, and a space to cook • Offer a place for women to bathe • Increase the space (for high volume facilities)
	Enhance cultural acceptability	• Restrict mixing of pregnant women with long-term patient families, neonates, or travelers in the same room • Work to dispel misconceptions surrounding shelter • Continue to sensitize male spouses • Improve individual planning and estimates of last menstrual period
	Increase continuity of care	• Provide routine health visits, well baby classes • Provide skills classes to build capacity and prevent boredom
**2. Improve governance and accountability structure**	Provide oversight and general governance	• Facilitate community to elect a Shelter Steering Committee likely consisting of Headmen, NHC members, facility staff or other community leaders. • Facilitate governance committee to establish bylaws
	Create an accountability framework for daily operations	• Facilitate the community to elect a shelter management individual or team (likely to vary by community) to work closely with facility staff and manage daily operations. • Detail the roles and expectations of Headmen and community groups, facility staff, government officials, and women and their families • Ensure clinic staff are technically involved for clinical work and oversight, but not burdened with daily operations
	Monitor shelter operations and evaluate its success	• Establish clear operating protocols • Develop metrics for success that meet the needs of all stakeholders from Government to traditional leadership, to community members • Develop guidelines to oversee that contributions and operations are happening according to plan.
**3. Build on existing efforts to foster sustainability through community ownership and individual responsibility**	Improve community sensitization	• Ensure facility messages to community members are clear and consistent • Strengthen programs coordinated by Chiefs and Headmen supporting birth planning, prevention of early marriage, and other women and girls’ health initiatives to improve planning • Build upon the continued work of community promotors for referrals, promoting skilled delivery, and empowering women and families to improve financial planning for birth • Continue community sensitization efforts with consistent messaging through other programs • Work with Community Development Fund, and other partners to improve community’s ability to build and maintain physical structure.

The governance and management structure responds directly to community suggestions to improve the oversight and daily operations of the MWHs. A governance committee would set the policies and procedures of the MWH and oversee its long-term sustainability. The management structure would consist of one or more individuals who oversees daily operations of the MWH and would be adaptable to meet the needs at different sites, e.g. using existing health facility staff, volunteers, or paid staff. This governance and management structure developed from community and stakeholder input is meant to be flexible enough to be implemented in different communities.

Because respondents told us that communities, government, and traditional leadership should have a role in sustainability, we specifically developed ideas for how all parties could contribute. Combining our study findings and the sustainability framework, we constructed a four-pronged financial sustainability strategy. The first three prongs were informed by the overarching sustainability framework constructs of internal and external sources of revenue, and in-kind contributions [1]. Internal sources of revenue include a budget line item in facility and district budgets and MWHs on the agenda for district and provincial strategic planning to demonstration government commitment; and engagement of traditional leadership to collect village taxes for the MWH. External sources of revenue include a social enterprise that serves the dual purpose of meeting a community need and generating revenue for the MWH. In-kind includes contributions of labor and materials from community members for construction, maintenance and cleaning the MWHs. The fourth prong of the financial sustainability strategy is financial literacy of those overseeing the MWH. The financial literacy prong includes training in developing a business plan for the social enterprise, as well as training in bookkeeping, cost projections, and evidence-based decision making.

It is not viable to rely on donor funding for the yearly recurrent costs of the MWH, such as maintenance, purchase of supplies and equipment, or stipends for the manager. Furthermore, no single source of local funding is likely to sustain the MWH operations long-term. For these reasons, we developed the model and its associated financial sustainability strategy to function independent of continued donor funding, within a resource-limited environment. While initial construction requires a large investment, with the management structure and financial strategy in place, the maternity waiting homes have the potential to be operationally and financially sustainable. This proposed model addresses not only the problems cited by our respondents, but many of the structural, cultural and financial challenges to MWHs identified in previous studies [23–25]. This model has been adapted to incorporate other similar findings [18] and is currently being implemented and evaluated for effectiveness and sustainability.

### Strengths and limitations

This was a cross-sectional design consisting of mixed, but primarily qualitative methods, so results cannot speak to changes over time nor are they generalizable to all of rural Zambia. However, the general approach for designing an intervention, inclusive of formative research, stakeholder engagement, and extensive community input could be applied widely. This study used a rigorous approach grounded in theory which has utility for public health researchers and program evaluators. It serves an example for readers of how to design a formative research study to generate findings that can inform design of an intervention for sustainability.

## Conclusions

In Southern Province, Zambia, MWHs appear to be a feasible and acceptable strategy to improve uptake of facility-based deliveries and subsequently improve maternal and child health outcomes. By developing their own solutions from the ground up, communities foster a sense of ownership and commitment that might increase the likelihood of operational and financial sustainability. With this in mind, our formative evaluation methods were chosen to empower communities to identify their problems, needs, and solutions with respect to access to and utilization of safe delivery. It is essential to rigorously test and evaluate models developed with this approach for sustainability.

## Supporting information

S1 Fig Conceptual framework for sustainability of public health programs. This conceptual framework was used to guide the development of this study [1].
(PDF)

S1 File Free Listing study instruments. These are the instruments used for the Free Listing and household survey for women, men, and elders, in English and the local language of Tonga.
(PDF)

S2 File Focus group discussion guide. This is the study instrument used for the focus group discussions with women, men, TBAs/SMAGs, and mothers-in-law, in English and the local language of Tonga.
(PDF)

S3 File Key informant interview guide. This is the study instrument used for the key informant interviews, in English and the local language of Tonga.
(PDF)

S4 File Maternity waiting home costing tool. This is the tool used for analyzing the fixed and variable costs of a maternity waiting home.
(XLSX)

S1 Dataset Free list dataset. This is the dataset containing the aggregate pile sorting totals for women, men, and elders who responded to the three free list questions.
(XLSX)
