# Supplementary material for: Listening to the community: Using formative research to strengthen maternity waiting homes in Zambia
Source: PLoS One. 2018 Mar 15;13(3):e0194535. doi: 10.1371/journal.pone.0194535 (PMC5854412; doi:10.1371/journal.pone.0194535)
Supplement: S1 Fig — This conceptual framework was used to guide the development of this study [1]. (PDF) [file pone.0194535.s001.pdf]

Conceptual framework for sustainability of public health programs

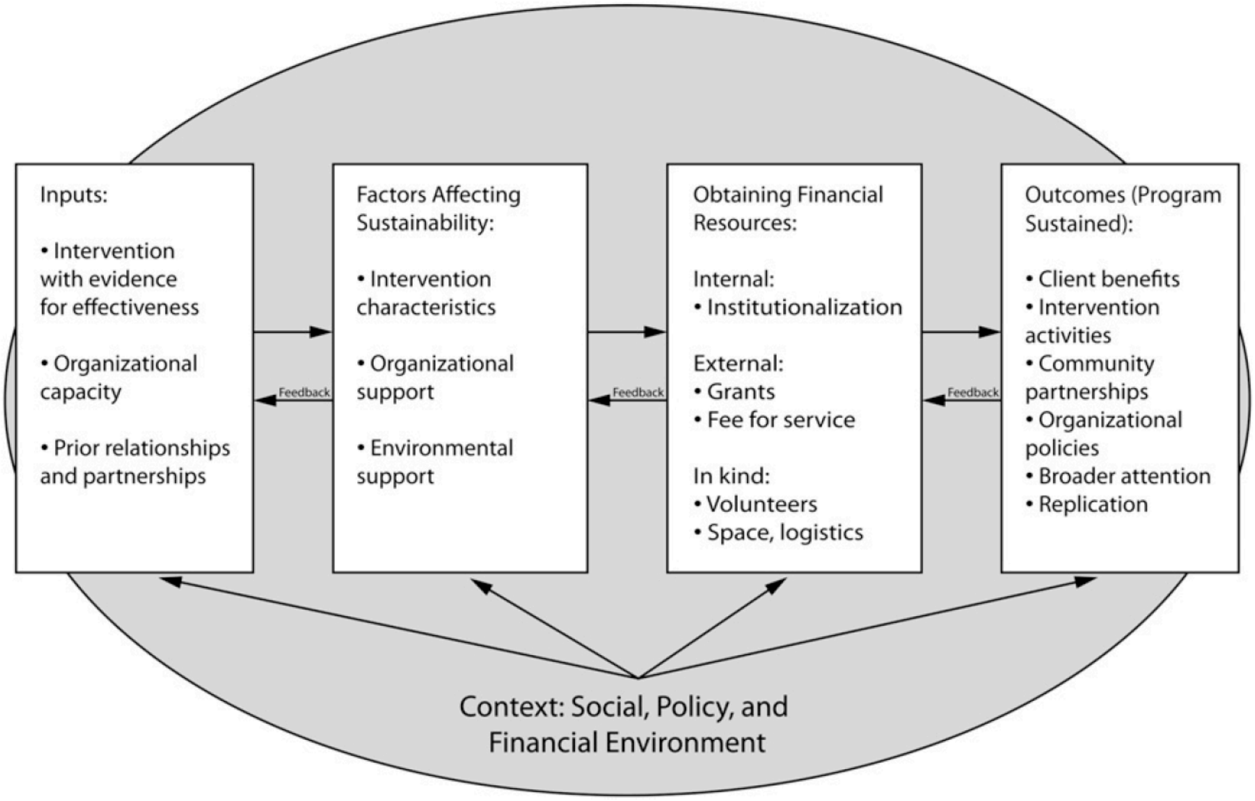

Source: Scheirer MA, Dearing JW. An agenda for research on the sustainability of Public Health Programs. Am J Public Health. 2011; 101: 2059-2067.
