# Supplementary material for: Listening to the community: Using formative research to strengthen maternity waiting homes in Zambia
Source: PLoS One. 2018 Mar 15;13(3):e0194535. doi: 10.1371/journal.pone.0194535 (PMC5854412; doi:10.1371/journal.pone.0194535)
Supplement: S1 File — These are the instruments used for the Free Listing and household survey for women, men, and elders, in English and the local language of Tonga. (PDF) [file pone.0194535.s002.pdf]

INSTRUMENT - FL_1:

Free Listing for recently delivered/pregnant women **SURVEY ID**

Instrument ID: FL_1

Sustainable Access for Waiting Mothers in Zambia

Free Listing Instrument

Target Audiences:

1)Recently Delivered/Pregnant Women

Instructions for the Interviewer

Step 1: Informed Consent: *Ask the participant for a few minutes of their time. Introduce yourself and the study. Begin the informed consent as per the training. If consent is granted, leave the informed consent sheet with the participant.*

Was verbal informed consent obtained?

YES ________ (proceed with interview)

NO ________ (STOP! Thank the participant for their time but do not proceed with the interview)

Interviewer: *Read the following statement. Please repeat the statement translated into the local language based on primary languages used by the group.*

"Thank you for agreeing to participate in this interview. My name is ______. I will be asking you the questions. My partner ______ will be taking notes on the things you have to say.

We want to understand your views on pregnancy and delivery in your community. To make these mothers' shelters sustainable, it might be necessary for mothers or other community members to pay something to stay at the shelter to keep them functioning. Please feel free to tell us whatever you are comfortable sharing. You should also remember that you do not have to share anything that you are not comfortable sharing. We will not write down your name. There are no right or wrong answers, so please be honest and tell us what is true for you and your community. Are you ready to begin?"

Step 2: *Please begin the interview with the demographic questions.*

Step 3: *Proceed to the Free Listing exercise. For the Free Listing exercise, please probe to obtain as many problems as possible for each question. When the participant cannot think of anything else, the list is completed. Please then return to the top of the list and ask for a short description of each problem. In the last step, please review the list and descriptions and mark each problem with a name or description. For each problem, please ask who in the community deals with the issue or is knowledgeable about it. Record the name and address of the knowledgeable persons identified.*

Step Four: *Proceed to the 'Willingness to Pay' questions. You will be asking respondents a set of questions to determine how much the mothers' shelter should charge and how to make them sustainable.*

Interviewer ID _________ **Note-taker ID:________________**

1. **Interview Date (DD/MM/YYYY)** ___________________
2. **Time Start** _______ **Time Finish** ___________
3. **Supervisor initials** __________

INSTRUMENT - FL_1:

Free Listing for recently delivered/pregnant women **SURVEY ID**

Table 1: Respondent Demographics

Interviewer: *"I'm going to start by asking you questions about yourself, your household and your pregnancies."*

Q#	QUESTION	CODE	Response	SKIP
0000	Distance from Facility	Less than 5 km (1)<br>5-10 km (2)<br>Greater than 10 km (3)		
000	Respondent/Instrument type	Women FL_1: **(1)**<br>Men FL_2: **(2)**<br>Elders FL_3: **(3)**	**1**	
100.	Respondent gender	Male **(1)**<br>Female **(2)**	**2**	
101.	What catchment area do you live in?	Naluja **(1)**<br>Kanchele **(2)**<br>Batoka **(3)**<br>Mapanza **(4)**	**4**	
102.	How old were you at your last birthday?	Years (_______)	__________	
103.	What is your marital status?	Married/in union **(1)**<br>Widowed,separated,divorced **(2)**<br>Single, never married **(3)**	_____________	
104.	How many living children do you have?	None **000**<br>Number ___		
105.	How many children, yours or others, under 18 live in your household?	None **000**<br>Number __	_____________	
106.	How many adults (including yourself) over the age of 18 live in your household?	None **000**<br>Number __		
107.	Do you work outside the home?	No **(0)**<br>Yes **(1)**		
108.	Mothers' shelters are homes where a pregnant woman can stay before she delivers, in order to be close to a facility which offers skilled obstetric care, a place that is safe and can manage an emergency. Had you ever heard of mothers' shelters before now?	No **(0)**<br>Yes **(1)**	__________	
109.	**Tell me how much you agree with statements:**<br>Mothers' shelters are very important for the health of mothers and newborns	**(1)** Strongly agree<br>**(2)** Agree<br>**(3)** Neither agree nor disagree<br>**(4)** Disagree<br>**(5)** Strongly disagree	__________	
110.	It is very worthwhile for someone like me to stay in a Mothers' shelter during the last weeks of a pregnancy.		__________	
111.	What is the name of the nearest health facility to where you live?	Name: _________<br>Unknown: 999	_______________	
112.	How many times have you been pregnant?	Number _____	__________	
113.	How many live children have you delivered?	None **000**<br>Number___	__________	
114.	Where was your last child delivered?	**(1)** In a home<br>**(2)** Health facility<br>**(3)** Hospital<br>**(4)** Other (specify)	_______________	
115.	Are you currently pregnant?	No **(0)**<br>Yes **(1)**	__________	**If No, skip to 117**

INSTRUMENT - FL_1:

Free Listing for recently delivered/pregnant women **SURVEY ID**

116.	Today, where do you think you will deliver this baby?	**(1)** In a home<br>**(2)** Health Facility<br>**(3)** Hospital<br>**(4)** Other (specify)	___________	
117.	Have you ever been told that your pregnancy was "high risk"?	No **(0)**<br>Yes **(1)**<br>Don't know **(99)**	________	If yes, what did you do?
118.	Do you know where your baby could be delivered using skilled obstetric care, a place that is safe and can manage an emergency?	No **(0)**<br>Yes **(1)**	___________	**If No skip to 122.**
119.	What is the name of the nearest facility where you could receive skilled obstetric care, a place that is safe and can manage an emergency?	Name____________<br>Don't know **(99)**	________________	
120.	How would you travel from your home to the nearest facility where you could receive skilled obstetric care, a place that is safe and can manage an emergency?	(1) By Foot<br>(2) By Taxi<br>(3) By Car<br>(4) By Bicycle<br>(5) By Ox cart<br>(6) Other (explain)		
121.	How long would it take you to travel from your home to the nearest facility, using the method of transportation that you just mentioned?	(1) Less than one hour<br>(2) More than one hour<br>(3) Over two hours		
122.	Do you want more children?	No **(0)**<br>Yes **(1)**<br>Don't know **(99)**	___________	
123.	If you were to deliver at a facility, how many days after delivery would you like to stay there? (if less than 1 day write hours)	Number day(s) ____<br>Number hour(s) ____	_________	
124.	If you were to stay in a mother's shelter, what services would you like to have offered to you at the Mothers' shelter after delivery?<br>*Select all that apply.*<br>*(do not read out the answers, let the participant offer answers and gently probe if necessary)*	**(1)** Post-Partum midwife visit for mom<br>**(2)** Continued pre-natal vitamins for mother<br>**(3)** 6 day newborn well-baby check<br>**(4)** Newborn care classes<br>**(5)** Family planning visit<br>**(6)** Other (specify)	_________<br>_________<br>_________<br>________<br>_________<br>_________<br>SPECIFY:	

INSTRUMENT - FL_1:

Free Listing for recently delivered/pregnant women **SURVEY ID**

Free Listing Exercises: ***Interviewer:*** *"Now I'm going to ask you questions about what people in your community think about certain issues. Let's get started. Please list as many things that come to your mind as you'd like."*

A. What are the biggest problems for pregnant women through delivery in the community?

Table 2: Problems	Problem Description	Who in the community deals with the issue or is knowledgeable about it? (Name, title, Phone and Address)
1.		
2.		
3.		
4.		
5.		
6.		
7.		
8.		
9.		
10.		

C) Tell me what pregnant woman in your community know or believe about mothers' shelters at health facilities?

Table 4. Knowledge or Belief	Description	Who in the community deals with the issue or is knowledgeable about it? (Name, title, Phone and Address)
1.		
2.		
3.		
4.		
5.		
6.		
7.		
8.		
9.		
10.		

INSTRUMENT - FL_1:

Free Listing for recently delivered/pregnant women **SURVEY ID**

D) What businesses or services are needed but not currently available in your community?

Table 5. Business or services	Description	Who in the community deals with the issue or is knowledgeable about it? (Name, title, Phone and Address)
1.		
2.		
3.		
4.		
5.		
6.		
7.		
8.		
9.		
10.		

INTERVIEWER: "Thank you sincerely for your time. We have completed this interview and are grateful for your help as we work to develop or improve on access to skill deliveries in your community." RETURN TO THE COVER PAGE AND NOTE THE TIME THE INTERVIEW WAS COMPLETED.

ADDITIONAL NOTES OR COMMENT FROM THE INTERVIEWER:

INSTRUMENT - FL_1:
Free Listing for recently delivered/pregnant women **SURVEY ID**

TONGA-Do not write responses on this form

Instrument ID: FL_1

Kuzumanana kuba abusena bwa kulindilila ba matumbu mucisi ca Zambia

Kwangunuka akulyaba

Beleela:

1)Batumbuka eno-eno / baala mada.

Malailile kuli sikubuzya

Ntaamu 1: Cizuminano: *Amubuzye sikotola lubazu kwatuzuzumina tusyoonto ku ciindi cabo. Amulipandulule alimwi abumvwuntauzyi. Amutalike a cizuminano mbuli mbokuyisigwa. Na bazumina, amubape cipepa cacizuminano. Batola lubazu.*

Sena bazunina kutole lubazu kwiinda mukwambaula?

Iyi _________ (zumanana amubandi)

Peepe _________ (amubalumbe basikutola lubazu kuciindi cabo pesi mutazumanani amubandi)

Sikubuzya : *Bala kaambo katobela.. Twakomba amwiindiluke kaambo mumulaka ngoba mvwa ba mukabunga.*

"Twalumba kuti mwakazumina kutola lubazu mumumbandi ooyu. Izina lyangu ndime _______. Ndilamubuzya mibuzyo. Mweenzuma _______ unolemba zyomunoowaamba.

Tuyanda kuziba mbomuyeya kulanganya twaambo twamada akutumbuka kubusena nkomukkala. Kucita kuti maanda akulindilila bamatumbu azumanane, inga cayandika kuti bamatumbu nokuba bantu bamucooko kubbadela kusyonto kukkala mung'anda yabamatumbu kutegwa kaabeleka. Amulimwvwe kwanguluka kutwambila alimwi kufumbwa ncomuyanda kutwambila.

Alimwi mweleede kuziba kuti tamweleede kwaamba zintu zomutalimvwi kwanguluka kwaamba. Tatukalembi izina lyenu. Kunyina bwinguzi butali kabotu na bulikabotu, twakomba amusyomeke kutwambila camansimpe lwenu akuli basimukobonyoko, sena mwalibambila tutalike? "

Ntaamu 2: *Twalomba amutalike kubuzya mibuzyo yabukkale .*

Ntaamu 3: Amuzumanane, kwaambaula kwanguluka kulemba zitobela twalomba kuti mubuzisisye kujana bwini kutwaambo tunji tukatazya kumubuzyo amubuzyo. Na sikwingula kwina cimbincayeeya, zyakwaamba zyamana. Amupilukile kumalembe akutalika akubuzya makatazyo ajanika. Kuntaamu ya mamanino, amwiinduluke nzyomwali kukanana abupanduluzyi akuzyiba zyina lyamakatazyo nokuba bupanduluzyi. Kupenzi apenzi, amubuzye muntu ukonzya kumana penzi nokuba kuba aaluzyibo. Amulembe zyina lyakwe akkeyala yamuntu ooyo.

Ntaamu 4: Amuzumanane kumibuzyo yakulyaba kubbadela. Munobuzya sikuyingula mibuzyo kuziba na maanda akulindilila bamatumbu anobbadelwa kucita kuti azumanane.

Interviewer ID __________ **Note-taker ID:_________________**

1. **Interview Date (DD/MM/YYYY)** ____________________
2. **Time Start** ________ **Time Finish** ____________
3. **Supervisor initials** ___________

INSTRUMENT - FL_1:
Free Listing for recently delivered/pregnant women **SURVEY ID**

TONGA-Do not write responses on this form

Table 1: Respondent Demographics
Sikubuzya: *"ndilamubuzya mibuzyo imugama, yang'anda yenu ayamada enu."*

Q#	QUESTION	CODE	Response	SKIP
0000	Distance from Health Facility	Less than 5 km (1)<br>5 to 10 km (2)<br>Greater than 10 km (3)		
000	Sikuingula / cibelesyo	Bakaintu FL_1: **(1)**<br>Balumi FL_2: **(2)**<br>Bapati FL_3: **(3)**	______________	
100.	Sikuingula mwalumi/mukaintu	Musankwa **(1)**<br>Musimbi **(2)**	______________	
101.	Mukkala kucooko cili?	Naluja **(1)**<br>Kanchele **(2)**<br>Batoka **(3)**<br>Mapanza **(4)**	______________	
102.	Mwakali a myaka yongaye mwakali?	Myaka (___ ____)	______ _______	
103.	Sena mulikwetwe?	Kukwatwa/ kukkalatomwe (1)<br>kufwidwa,Kwanzana,kulekana **(2)**<br>Nabutema **(3)**	______________	
104.	Muli abana bongaye bapona?	Kunyina **000**<br>Nambala ___		
105.	Kuli bana bongaye benu na bakulela bata siki myaka ili kumi alusele (18) bakkala mung'anda yenu ?	Kunyina **000**<br>Nambala __	______________	
106.	Balibongaye bapati (kuvwela andinywe) balamyaka iinda ikkumi alusele (18) bakkala ang'anda yenu?	Kunyina **000**<br>Nambala __		
107.	Sena mulabeleka?	Peepe **(0)**<br>Iyi **(1)**		
108.	Maanda abamatumbu maanda akulindilila ba simada kabatanatumbuka kuti babe afwafwi azibbadela zijisi basiabupampu mukutumbusya ciindi cakutumbuka casika. Senakuli nomwakamvide maanda aya ?	Peepe **(0)**<br>Iyi **(1)**	__________	
109.	**Mundambile mbomuzumina ku twaambo atu:**<br>Maanda abamatumbu alayandika kapati ku nseba zyabamatumbu abana bamvwanda	**(1)** Ndilazumina kapati<br>**(2)** Ndilazumina<br>**(3)** Ndilazumina/ tandizumini<br>**(4)** Ndilakaka<br>**(5)** Ndilakaka kapati	__________	
110.	NCibotu kapati kumuntu mbuli ndime kukkala mumaanda abamatumbu kwacaala vwiki zisyoonto zya kutumbuka.		__________	
111.	Ndizinanzi lyacibbadela / kabbadela cili munisi-munsi ankomukkala?	Izina: _________<br>Tabazyi: 999	_______________	
112.	Mu mitidezindi zyongaye?	Nambala _____	__________	
113.	Ino balibo ngaye bana mbomwakatumbuka kabali bazumi (kabayoya)?	Kunynia **000**<br>Nambala___	________________	

INSTRUMENT - FL_1:
Free Listing for recently delivered/pregnant women **SURVEY ID**

TONGA-Do not write responses on this form

114.	Ino mwana wenu mulesi mwamutumbukila kuli?	**(1)** Kunganda<br>**(2)** Kukabbadela<br>**(3)** Kucibbadela<br>**(4)** Kumwi (kugame)	________________	
115.	Sena mulimisi?	Peepe **(0)**<br>Iyi **(1)**	__________	**Na Peepe koya ku 117**
116.	Sunu, muyeya kuti muyotumbukila kuli?	**(1)** Kunganda<br>**(2)** Kabbadela<br>**(3)** Kucibbadela<br>**(4)** Kumwi (kugame)	__________	
117.	Sena kuli nomwakambilwa kuti "kuli buyumu yumu"bupati ku dal yenu?	Peepe **(0)**<br>Iyi **(1)**<br>Sezyi **(99)**	Na Iyi, mwakacita buti?<br>__________	
118.	Sena kuli nkomuzyi komunga mwatumbukila mwana wenu kuli kutumbusya kabotu?	Peepe **(0)**<br>Iyi **(1)**	__________	**Na Peepe koya ku 122**
119.	Mbusena nzi bwakabbadela / cibbadela bulaafwafwi nkomukonzya kujana bakonzya kumutumbusya kabotu balaluzyibo?	Izina___________<br>Sezyi **(99)**	________________	
120.	Mwenda buti kuzwa komukkala kusika kukabbadela / cibbadela komunga mwajana lugwasyo kuzwakuliba syaabupampu batumbusya?	(1) Amaulu<br>(2) Amota ya kubbadela<br>(3) Amota<br>(4) Ancinga<br>(5) Acikochi<br>(6) Cinwi (cigame)		
121.	Cimutolela ciindi cilamfwu buti kuzwa komukkala kusika kubusena bula fwafwi kubelesya ceenzyo comwaamba?	(1) Kutasika woola lyoomwe<br>(2) Kwinda woola lyoomwe<br>(3) Kwinda mawoola obilo		
122.	Muciyanda bana bambi?	Peepe **(0)**<br>Iyi **(1)**<br>Sezyi **(99)**	__________	
123.	Namwatumbukila kukabbadela / cibbadela muyanda kukkala mazuba ongaye mwamana kutumbuka?	Nambala____	________	
124.	Nakutimwakala mung'anda yaba matumbu cinzi ncomuyanda kuti kabamucitila mwamana kutumbuka?<br><br>*Sala zyoonse zyeelede.*	**(1)** Kulangwalangwa asiku tumbusya mwamana kutumbuka<br>**(2)** Kuzumanana kunwa ma pilusi avitameni ada.<br>**(3)** Kulangwa kwa mwana mumvwanda kwainda mazuba ali osanwe akamwi akuyiisigwa<br>**(4)** Kuyiya kubamba mwana mumvwanda<br>**(5)** Kutantanya bana (family planning)<br>**(6)** Zimwi (zigame)	________<br>________<br>________<br>________	

INSTRUMENT - FL_1:
Free Listing for recently delivered/pregnant women **SURVEY ID**

TONGA-Do not write responses on this form

Free Listing Exercises: ***Sikubuzya:*** *"eno ndiyanda kumubuzya mibuzyo mbuli mbo bayeeya bantu kucooko cenu munzintu zimwi. Atutalike. Twalomba mwaambe zintu zyoonse zyili kumoyokwenu.*

A. Mapenzi nzi mapati kuli bamakaintu bamada kucooko cenu?

Table 2: Mapenzi	Musyobo wamapenzi	Nguniwendelezya na ulaluzibo kumapenzi aya ? (izina cacita, nambala ya foni akubusena nkwakkala)
1.		
2.		
3.		
4.		
5.		
6.		
7.		
8.		
9.		
10.		

C) Mundambile eco bantu mucooko cenu cobazyi na cobasyoma kujatikizya maanda aba matumbu? (buzisisye kujana bwini bwa milawo na tunsia-nsia aluymwi zitobelwa, busena akwiindana kwa luzibo akati ka baalumi abamaintu alimwi a bapati / (bamacembele)

Table 4. Luzibo na lusyomo	Bupanduluzi	Nguniwendelezya na ulaluzibo kumapenzi aya ? (izina cacita, nambala ya foni akubusena nkwakkala)
1.		
2.		
3.		
4.		
5.		
6.		
7.		

8.		
9.		
10.		

D) Makwebonzi na ncito ziyandikana zitako kucooko cenu?

Table 5. Makwebo Na Ncito	Musyobo Wamakwebo	Nguniwendelezya na ulaluzibo kumapenzi aya ? (izina cacita, nambala ya foni akubusena nkwakkala)
1.		
2.		
3.		
4.		
5.		
6.		
7.		
8.		
9.		

Sikubuzya: "Ndalumba kapati ku ciindi cenu. Twamana mubandi oyu alimwi tuli lumbide kugwasya kwenu mbotuyobeleka kubamba na ku ba mbulula maanda abamatumbu mu cooko cenu." PILUKA KU PEPA LYA KUSANGUNA A KULEMBA CIINDI MUBANDI NOWAMANA.

INSTRUCTION - FL_2:
Free Listing for Men with children <2

SURVEY ID

Instrument ID: FL_2

Sustainable Access for Waiting Mothers in Zambia

Free Listing Instrument

Target Audiences:

1)Men with children 2 years of age or younger

Instructions for the Interviewer

Step 1: Informed Consent: *Ask the participant for a few minutes of their time. Introduce yourself and the study. Begin the informed consent as per the training. If consent is granted, leave the informed consent sheet with the participant.*

Was verbal informed consent obtained?

YES _________ (proceed with interview)

NO _________ (STOP! Thank the participant for their time but do not proceed with the interview)

***Interviewer**: Read the following statement. Please repeat the statement translated into the local language based on primary languages used by the group.*

"Thank you for agreeing to participate in this interview. My name is ______. I will be asking you the questions. My partner _______ will be taking notes on the things you have to say.

We want to understand your views on pregnancy and delivery in your community. To make these mothers' shelters sustainable, it might be necessary for mothers or other community members to pay something to stay at the shelter to keep them functioning. Please feel free to tell us whatever you are comfortable sharing. You should also remember that you do not have to share anything that you are not comfortable sharing. We will not write down your name. There are no right or wrong answers, so please be honest and tell us what is true for you and your community. Are you ready to begin?"

Step 2: *Please begin the interview with the demographic questions.*

Step 3: *Proceed to the Free Listing exercise. For the Free Listing exercise, please probe to obtain as many problems as possible for each question. When the participant cannot think of anything else, the list is completed. Please then return to the top of the list and ask for a short description of each problem. In the last step, please review the list and descriptions and mark each problem with a name or description. For each problem, please ask who in the community deals with the issue or is knowledgeable about it. Record the name and address of the knowledgeable persons identified.*

Step Three: *Proceed to the 'Willingness to Pay' questions. You will be asking respondents a set of questions to determine how much the mothers' shelter should charge and how to make them sustainable.*

Interviewer ID __________ **Note-taker ID:_________________**

1. **Interview Date (DD/MM/YYYY)** ____________________
2. **Time Start** ________ **Time Finish** ____________
3. **Supervisor initials** ___________

INSTRUMENT - FL_2:
Free Listing for Men with children <2 **SURVEY ID** [ ]

Table 1: Respondent Demographics
Interviewer: *"I'm going to start by asking you questions about yourself, your household and your wife's pregnancies."*

Q#	QUESTION	CODE	Response	SKIP
0000	Distance from Facility	Less than 5 km (1)<br>5-10 km (2)<br>Greater than 10 km (3)		
000.	Respondent/Instrument type	Women FL_1: **(1)**<br>Men FL_2: **(2)**<br>Elders FL_3: **(3)**	**2**	
100.	Respondent gender	Male **(1)**<br>Female **(2)**	**1**	
101.	What catchment area do you live in?	Naluja **(1)**<br>Kanchele **(2)**<br>Batoka **(3)**<br>Mapanza**(4)**	**4**	
102.	How old were you at your last birthday?	Years (___ ____)	_____________	
103.	What is your marital status?	Married/in union **(1)**<br>Widowed,separated,divorced **(2)**<br>Single, never married **(3)**	__________	# of wives:
104.	How many living children do you have?	None **000**<br>Number ___	__________	
105.	How many children, yours or others, under 18 live in your household?	None **000**<br>Number __	_____________	
106.	How many adults (including yourself) over the age of 18 live in your household?	None **000**<br>Number __		
107.	Do you work outside the home?	No **(0)**<br>Yes **(1)**		
108.	Mothers' shelters are homes where a pregnant woman can stay before she delivers, in order to be close to a facility which offers skilled obstetric care, that is safe and can manage an emergency.<br>Had you ever heard of Mothers' shelters before now?	No **(0)**<br>Yes **(1)**	__________	
109.	**Tell me how much you agree with statements:**<br>Mothers' shelters are very important for the health of mothers and newborns	**(1)** Strongly agree<br>**(2)** Agree<br>**(3)** Neither agree nor disagree<br>**(4)** Disagree<br>**(5)** Strongly disagree	__________	
110.	It is very worthwhile for someone like my wife to stay in a Mothers' shelter during the last weeks of a pregnancy.		__________	
111.	What is the name of the nearest health facility to where you live?	Name: _________<br>Unknown: 999	______________	
112.	How many times has your wife been pregnant? (if multiple wives, total number)	Number _____	_______	
113.	How many live children has your wife delivered?<br>(if multiple wives, total number)	None **000**<br>Number___	__________	
114.	Where was your last child delivered?	**(1)** In a home<br>**(2)** Health facility<br>**(3)** Hospital<br>**(4)** Other (specify)	______________	

INSTRUMENT - FL_2:
Free Listing for Men with children <2 **SURVEY ID**

115.	Is your wife/spouse/partner currently pregnant? (if multiple wives, ask if any are pregnant)	No **(0)**<br>Yes **(1)**<br>Don't know **(99)**	__________	**If No, skip to 117**
116.	Today, where do you think your wife will deliver this baby?	**(1)** In a home<br>**(2)** Health Facility<br>**(3)** Hospital<br>**(4)** Other (specify)	__________	
117.	Has your wife (wives) ever been told that her pregnancy was "high risk"?	No **(0)**<br>Yes **(1)**<br>Don't know **(99)**		If yes, what did you do?
118.	Do you know where your baby could be delivered using skilled obstetric care, a place that is safe and can manage an emergency?	No **(0)**<br>Yes **(1)**	__________	**If No skip to 122.**
119.	What is the name of the nearest facility where your wife could receive skilled obstetric care, a place that is safe and can manage an emergency?	Name___________<br>Don't know **(99)**	_____________	
120.	How would your wife travel from your home to the nearest facility where she could receive skilled obstetric care, a place that is safe and can manage an emergency?	(1) By Foot<br>(2) By Taxi<br>(3) By Car<br>(4) By Bicycle<br>(5) By Ox cart<br>(6) Other (explain)		
121.	How long would it take you to travel from your home to the nearest facility, using the method of transportation that you just mentioned?	(1) Less than one hour<br>(2) More than one hour<br>(3) Over two hours		
122.	Do you want more children?	No **(0)**<br>Yes **(1)**<br>Don't know **(99)**	__________	
123.	If your wife were to deliver at a facility, how many days after delivery would your wife like to stay there? (if less than 1 day write hours)	Number day(s) ____<br>Number hour(s) ____	________	
124.	If your wife were to stay in a mother's shelter, what services would you like to have offered to her at the Mothers' shelter after delivery?<br><br>*Select all that apply.*<br><br>*(do not read out the answers, let the participant offer answers and gently probe if necessary)*	**(1)** Post-Partum midwife visit for mom<br>**(2)** Continued pre-natal vitamins for mother<br>**(3)** 6 day newborn well-baby check<br>**(4)** Newborn care classes<br>**(5)** Family planning visit<br>**(6)** Other (specify)	________<br>________<br>________<br>________<br>________<br>________<br>SPECIFY:	

INSTRUMENT - FL_2:

Free Listing for Men with children <2 **SURVEY ID**

Free Listing Exercises: ***Interviewer:*** *"Now I'm going to ask you questions about what people in your community think about certain issues. Let's get started. Please list as many things that come to your mind as you'd like."*

A. What are the biggest problems for pregnant women through delivery in the community?

Table 2: Problems	Problem Description	Who in the community deals with the issue or is knowledgeable about it? (Name, title, Phone and Address)
1.		
2.		
3.		
4.		
5.		
6.		
7.		
8.		
9.		
10.		

C) Tell me what men in your community know or believe about mothers' shelters?

Table 4. Knowledge or Belief	Description	Who in the community deals with the issue or is knowledgeable about it? (Name, title, Phone and Address)
1.		
2.		
3.		
4.		
5.		
6.		
7.		
8.		
9.		

10.		

D) What businesses and services are needed but not available in your community?

Table 5. Business or services	Description	Who in the community deals with the issue or is knowledgeable about it? (Name, title, Phone and Address)
1.		
2.		
3.		
4.		
5.		
6.		
7.		
8.		
9.		
10.		

INTERVIEWER: "Thank you sincerely for your time. We have completed this interview and are grateful for your help as we work to develop or improve on access to skill deliveries in your community." RETURN TO THE COVER PAGE AND NOTE THE TIME THE INTERVIEW WAS COMPLETED.

ADDITIONAL NOTES OR COMMENT FROM THE INTERVIEWER:

INSTRUMENT - FL_2:
Free Listing for men with children <2 **SURVEY ID**

TONGA-do not write responses on this form.

Instrument ID: FL_2

Kuzumanana kuba abusena bwa kulindilila ba matumbu mucisi ca Zambia

FL Men w/Children < 2

Beleela:

1) Men w/ Children < 2

Malailile kuli sikubuzya

Ntaamu 1: Cizuminano: *Amubuzye sikotola lubazu kwatuzuzumina tusyoonto ku ciindi cabo. Amulipandulule alimwi abumvwuntauzyi. Amutalike a cizuminano mbuli mbokuyisigwa. Na bazumina, amubape cipepa cacizuminano. Batola lubazu.*

Sena bazunina kutole lubazu kwiinda mukwambaula?

Iiyi ________ (zumanana amubandi)

Peepe ________ (amubalumbe basikutola lubazu kuciindi cabo pesi mutazumanani amubandi)

Sikubuzya *: Bala kaambo katobela.. Twakomba amwiindiluke kaambo mumulaka ngoba mvwa ba mukabunga.*

“Twalumba kuti mwakazumina kutola lubazu mumumbandi ooyu. Izina lyangu ndime ______. Ndilamubuzya mibuzyo. Mweenzuma ______ unolemba zyomunoowaamba.

Tuyanda kuziba mbomuyeya kulanganya twaambo twamada akutumbuka kubusena nkomukkala. Kucita kuti maanda akulindilila bamatumbu azumanane, inga cayandika kuti bamatumbu nokuba bantu bamucooko kubbadela kusyonto kukkala mung’anda yabamatumbu kutegwa kaabeleka. Amulimwvwe kwangunuka kutwambila alimwi kufumbwa ncomuyanda kutwambila.

Alimwi mweleede kuziba kuti tamweleede kwaamba zintu zomutalimvwi kwanguluka kwaamba. Tatukalembi izina lyenu. Kunyina bwinguzi butali kabotu na bulikabotu, twakomba amusyomeke kutwambila camansimpe lwenu akuli basimukobonyoko, sena mwalibambila tutalike? “

Ntaamu 2: *Twalomba amutalike kubuzya mibuzyo yabukkale .*

Ntaamu 3: Amuzumanane, kwaambaula kwanguluka kulemba zitobela twalomba kuti mubuzisisye kujana bwini kutwaambo tunji tukatazya kumubuzyo amubuzyo. Na sikwingula kwina cimbincayeeya, zyakwaamba zyamana. Amupilukile kumalembe akutalika akubuzya makatazyo ajanika. Kuntaamu ya mamanino, amwiinduluke nzyomwali kukanana abupanduluzyi akuzyiba zyina lyamakatazyo nokuba bupanduluzyi. Kupenzi apenzi, amubuzye muntu ukonzya kumana penzi nokuba kuba aaluzyibo. Amulembe zyina lyakwe akkeyala yamuntu ooyo.

Ntaamu 4: Amuzumanane kumibuzyo yakulyaba kubbadela. Munobuzya sikuyingula mibuzyo kuziba na maanda akulindilila bamatumbu anobbadelwa kucita kuti azumanane.

Interviewer ID _________ **Note-taker ID:________________**

1. **Interview Date (DD/MM/YYYY)** __________________
2. **Time Start** _______ **Time Finish** ___________
3. **Supervisor initials** __________

TONGA-do not write responses on this form.

INSTRUMENT - FL_2:

Free Listing for men with children <2 **SURVEY ID**

Table 1: Respondent Demographics

Sikubuzya: *"ndilamubuzya mibuzyo imugama, yang'anda yenu ayamada enu madaa abakaintu beenu"*

Q#	QUESTION	CODE	Response	SKIP
000	Sikuingula / cibelesyo	Bakaintu FL_1: **(1)**<br>Balumi FL_2: **(2)**<br>Bapati FL_3: **(3)**	______________	
100.	Sikuingula mwalumi/mukaintu	Musankwa **(1)**<br>Musimbi **(2)**	______________	
101.	Mukkalae kucooko cili?	Naluja **(1)**<br>Kanchele **(2)**<br>Batoka **(3)**<br>Mapanza **(4)**	______________	
102.	Mwakali a myaka yongaye mwakali?	Myaka (___ ____)	______ _______	
103.	Sena mulikwetwe?	Kukwatwa/kukkalatomwe **(1)**<br>Kufwidwa,Kwanzana, kulekana **(2)**<br>Nabutema **(3)**	Nombolo yabamakaintu<br>______________	
104.	Muli abana bongaye bapona?	Kunyina **000**<br>Nombolo ___		
105.	Kuli bana bongaye benu na bakulela bata siki myaka ili kumi alusele (18) bakkala mung'anda yenu ?	Kunyina **000**<br>Nombolo __	______________	
106.	Balibongaye bapati (kuvwela andinywe) balamyaka iinda ikkumi alusele (18) bakkala ang'anda yenu?	Kunyina **000**<br>Nombolo __		
107.	Sena mulabeleka?	Peepe **(0)**<br>Iiyi **(1)**		
108.	Maanda abamatumbu maanda akulindilila ba simada kabatanatumbuka kuti babe afwafwi azibbadela zijisi basiabupampu mukutumbusya ciindi cakutumbuka casika. Senakuli nomwakamvide maanda aya ?	Peepe **(0)**<br>Iiyi **(1)**	___________	
109.	**Mundambile mbomuzumina ku twaambo atu:**<br>Maanda abamatumbu alayandika kapati ku nseba zyabamatumbu abana bamvwanda	**(1)** Ndilazumina kapati<br>**(2)** Ndilazumina<br>**(3)** Ndilazumina/ tandizumini<br>**(4)** Ndilakaka<br>**(5)** Ndilakaka kapati	___________	
110.	Ncibotu kapati kumuntu mbuli bakaintu bangu kukkala mumaanda abamatumbu kwacaala vwiki zisyoonto zya kutumbuka.		___________	
111.	Ndizinanzi lyacibbadela / kabbadela cili munisi-munsi ankomukkala?	Izina: _________<br>Tabazyi: 999	________________	
112.	Ino bakaintu benu bakamita ziindi zyongaye?	Nombolo _____	___________	
113.	Ino balibo ngaye bana bakaintu benu mbobakatumuka kabali bazumi (kabayoya)? (Nabakaintu baji bali bongaye)	Kunynia **000**<br>Nombolo___	___________	
114.	Ino mwana wenu mulesi bamutumbukila kuli?	**(1)** Kunganda<br>**(2)** Kukabbadela<br>**(3)** Kucibbadela<br>**(4)** Kumwi (kugame)	________________	
115.	Sena bakaintu benu balimitide?	Peepe **(0)**		**Na**

TONGA-do not write responses on this form.

INSTRUMENT - FL_2:
Free Listing for men with children <2 **SURVEY ID**

	(Na bajisi bakaintu banji, buzya na kuli uumitide)	Iiyi **(1)**	___________	**Peepe koya ku 117**
116.	Sunu, muyeya kuti bakaintu benu bayo tumbukila kuli?	**(1)** Kunganda<br>**(2)** Kabbadela<br>**(3)** Kucibbadela<br>**(4)** Kumwi (kugame)	___________	
117.	Sena bakaintu benu kuli nobakambilwa kuti "kuli buyumu yumu" bupati ku da lyabo?	Peepe **(0)**<br>Iiyi **(1)**<br>Sezyi **(99)**	Na iiyi, mwakacita buti?<br>___________	
118.	Sena kuli nkomuzyi kobanga batumbukila mwana wenu kuli kutumbusya kubotu?	Peepe **(0)**<br>Iiyi **(1)**	___________	**Na Peepe koya ku 122**
119.	Mbusena nzi bwakabbadela / cibbadela bulaafwafwi mbobakonzya kujana bakonzya kumutumbusya kabotu balaluzyibo?	Izina____________<br>Sezyi **(99)**	________________	
120.	Beenda buti kuzwa komukkala kusika kukabbadela / cibbadela kobanga bajana lugwasyo kuzwakuliba syaabupampu batumbusya?	(1) Amaulu<br>(2) Amota ya kubbadela<br>(3) Amota<br>(4) Achiinga<br>(5) Acikochi<br>(6) Cinwi (cigame)		
121.	Cibatolela ciindi ciilamfwu buti kuzwa kobakkala kusika kubusena bula fwafwi kubelesya ceenzyo comwaamba?	(1) Kutasika woola lyoomwe<br>(2) Kwinda woola lyoomwe<br>(3) Kwinda mawoola obilo		
122.	Muciyanda bana bambi?	Peepe **(0)**<br>Iiyi **(1)**<br>Sezyi **(99)**	___________	
123.	Nabatumbukila kukabbadela / cibbadela muyanda kuti bakkale mazuba ongaye bamana kutumbuka?	Nombolo____	_________	
124.	Nakuti bakala mung'anda yaba matumbu cinzi ncomuyanda kuti kababacitila bamana kutumbuka?<br><br>*Sala zyoonse zyeelede.*	**(1)** Kulangwalangwa asiku tumbusya mwamana kutumbuka<br>**(2)** Kuzumanana kunwa ma pilusi avitameni ada.<br>**(3)** Kulangwa kwa mwana mumvwanda kwainda mazuba ali osamwe akamwi akuyiisigwa<br>**(4)** Kuyiya kubamba mwana mumvwanda<br>**(5)** Kutantanya bana (family planning)<br>**(6)** Zimwi (zigame)	_________<br>_________<br>_________<br>_________	

TONGA-do not write responses on this form.

INSTRUMENT - FL_2:
Free Listing for men with children <2 **SURVEY ID**

Free Listing Exercises: ***Sikubuzya:*** *"eno ndiyanda kumubuzya mibuzyo mbuli mbo bayeeya bantu kucooko cenu munzintu zimwi. Atutalike. Twalomba mwaambe zintu zyoonse zyili kumoyokwenu.*

A. Mapenzi nzi mapati kuli bamakaintu bamada kucooko cenu?

Table 2: Mapenzi	Musyobo wamapenzi	Nguniwendelezya na ulaluzibo kumapenzi aya ? (izina cacita, nambala ya foni akubusena nkwakkala)
1.		
2.		
3.		
4.		
5.		
6.		
7.		
8.		
9.		
10.		

C) Mundambile eco bantu mucooko cenu cobazyi na cobasyoma kujatikizya maanda aba matumbu? (buzisisye kujana bwini bwa milawo na tunsia-nsia aluymwi zitobelwa, busena akwiindana kwa luzibo akati ka baalumi abamaintu alimwi a bapati / (bamacembele)

Table 4. Luzibo na lusyomo	Bupanduluzi	Nguniwendelezya na ulaluzibo kumapenzi aya ? (izina cacita, nambala ya foni akubusena nkwakkala)
1.		
2.		
3.		
4.		
5.		
6.		
7.		
8.		

TONGA-do not write responses on this form.

9.		
10.		

D) Makwebonzi na ncito ziyandikana zitako kucooko cenu?

Table 5. Makwebo Na Ncito	Musyobo Wamakwebo	Nguniwendelezya na ulaluzibo kumapenzi aya ? (izina cacita, nambala ya foni akubusena nkwakkala)
1.		
2.		
3.		
4.		
5.		
6.		
7.		
8.		
9.		

Sikubuzya: "Ndalumba kapati ku ciindi cenu. Twamana mubandi oyu alimwi tuli lumbide kugwasya kwenu mbotuyobeleka kubamba na ku ba mbulula maanda abamatumbu mu cooko cenu." PILUKA KU PEPA LYA KUSANGUNA A KULEMBA CIINDI MUBANDI NOWAMANA.

INSTRUMENT - FL_3:
Free Listing for Community Elders

SURVEY ID **4-056**

Instrument ID: FL_3

Sustainable Access for Waiting Mothers in Zambia

Free Listing Instrument

Target Audiences:

1)Community Elders

Instructions for the Interviewer

Step 1: Informed Consent: *Ask the participant for a few minutes of their time. Introduce yourself and the study. Begin the informed consent as per the training. If consent is granted, leave the informed consent sheet with the participant.*

Was verbal informed consent obtained?

YES ________ (proceed with interview)

NO ________ (STOP! Thank the participant for their time but do not proceed with the interview)

***Interviewer**: Read the following statement. Please repeat the statement translated into the local language based on primary languages used by the group.*

"Thank you for agreeing to participate in this interview. My name is ______. I will be asking you the questions. My partner ______ will be taking notes on the things you have to say.

We want to understand your views on pregnancy and delivery in your community. To make these mothers' shelters sustainable, it might be necessary for mothers or other community members to pay something to stay at the shelter to keep them functioning. Please feel free to tell us whatever you are comfortable sharing. You should also remember that you do not have to share anything that you are not comfortable sharing. We will not write down your name. There are no right or wrong answers, so please be honest and tell us what is true for you and your community. Are you ready to begin?"

Step 2: *Please begin the interview with the demographic questions.*

Step 3: *Proceed to the Free Listing exercise. For the Free Listing exercise, please probe to obtain as many problems as possible for each question. When the participant cannot think of anything else, the list is completed. Please then return to the top of the list and ask for a short description of each problem. In the last step, please review the list and descriptions and mark each problem with a name or description. For each problem, please ask who in the community deals with the issue or is knowledgeable about it. Record the name and address of the knowledgeable persons identified.*

Step Three: *Proceed to the 'Willingness to Pay' questions. You will be asking respondents a set of questions to determine how much the mothers' shelter should charge and how to make them sustainable.*

Interviewer ID _________ **Note-taker ID:________________**

1. **Interview Date (DD/MM/YYYY)** ___________________
2. **Time Start** _______ **Time Finish** ___________
3. **Supervisor initials** __________

INSTRUMENT - FL_3:
Free Listing for Community Elders

SURVEY ID 4-056

Table 1: Respondent Demographics

Interviewer: *"I'm going to start by asking you questions about yourself, your household and your daughters/grandchild's pregnancies."*

Q#	QUESTION	CODE	Response	SKIP
0000	Distance from Facility	Less than 5 km (1)<br>5-10 km (2)<br>Greater than 10 km (3)		
000.	Respondent/Instrument type	Women FL_1: **(1)**<br>Men FL_2: **(2)**<br>Elders FL_3: **(3)**	**3**	
100.	Respondent gender	Male **(1)**<br>Female **(2)**	____________	
101.	What catchment area do you live in?	Naluja **(1)**<br>Kanchele **(2)**<br>Batoka **(3)**<br>Mapanza **(4)**	**4**	
102.	How old were you at your last birthday?	Years (___ ____)	____________	
103.	What is your marital status?	Married/in union **(1)**<br>Widowed,separated,divorced **(2)**<br>Single, never married **(3)**	____________	
104.	How many living children do you have?	None **000**<br>Number ___		
104. A	How many living grand-children do you have?	None **000**<br>Number ___		
105.	How many children/grandchildren, yours or others, under 18 live in your household?	None **000**<br>Number _____	____________	
106.	How many adults (including yourself) over the age of 18 live in your household?	None **000**<br>Number _____		
107.	Do you work outside the home?	No **(0)**<br>Yes **(1)**		
108.	Mothers' shelters are homes where a pregnant woman can stay before she delivers, in order to be close to a facility which offers skilled obstetric care, a place that is safe and can manage an emergency. Had you ever heard of mothers' shelters before now?	No **(0)**<br>Yes **(1)**	__________	
109.	**Tell me how much you agree with statements:** Mothers' shelters are very important for the health of mothers and newborns	**(1)** Strongly agree<br>**(2)** Agree<br>**(3)** Neither agree nor disagree<br>**(4)** Disagree<br>**(5)** Strongly disagree	__________	
110.	It is very worthwhile for someone like my daughter/daughter-in-law/granddaughter to stay in a mothers' shelter during the last weeks of a pregnancy.		__________	
111.	What is the name of the nearest health facility to where you live?	Name: _________<br>Unknown: 999	_____________	

[redacted]

114.	Where was your most recent child/grandchild delivered?	**(1)** In a home<br>**(2)** Health facility<br>**(3)** Hospital<br>**(4)** Other (specify)	______________	
115.	Are any of your children or grandchildren currently pregnant?	No **(0)**<br>Yes **(1)**<br>Don't know **(99)**	__________	**If No, skip to 117**
116.	Today, where do you think your child/grandchild will deliver this baby?	**(1)** In a home<br>**(2)** Health Facility<br>**(3)** Hospital<br>**(4)** Other (specify)	__________	
117.	Has any of your children/grandchildren ever been told that her pregnancy was "high risk"?	No **(0)**<br>Yes **(1)**<br>Don't know **(99)**		If yes, what did you do?
118.	Do you know where your child/grandchild's baby could be delivered using skilled obstetric care, a place that is safe and can manage an emergency?	No **(0)**<br>Yes **(1)**	__________	**If No skip to 122.**
119.	What is the name of the nearest facility where your child/grandchild could receive skilled obstetric care, a place that is safe and can manage an emergency?	Name___________<br>Don't know **(99)**	_____________	
120.	How would your child/grandchild travel from their home to the nearest facility where she could receive skilled obstetric care, a place that is safe and can manage an emergency?	(1) By Foot<br>(2) By Taxi<br>(3) By Car<br>(4) By Bicycle<br>(5) By Ox cart<br>(6) Other (explain)		
121.	How long would it take your child/grandchild to travel from their home to the nearest facility, using the method of transportation that you just mentioned?	(1) Less than one hour<br>(2) More than one hour<br>(3) Over two hours		
122.	Do you want more grandchildren?	No **(0)**<br>Yes **(1)**<br>Don't know **(99)**	__________	
123.	If your child/grandchild were to deliver at a facility, how many days after delivery would you like her to stay there? (if less than days, write hours)	Number day(s) ____<br>Number hour(s) ____	________	
124.	If she were to stay in a mother's shelter, what services would you like to have offered to her at the mothers' shelter after delivery?<br><br>*Select all that apply.*<br><br>*(do not read out the answers, let the participant offer answers and gently probe if necessary)*	**(1)** Post-Partum midwife visit for mom<br>**(2)** Continued pre-natal vitamins for mother<br>**(3)** 6 day newborn well-baby check<br>**(4)** Newborn care classes<br>**(5)** Family planning visit<br>**(6)** Other (specify)	________<br>_______<br>________<br>_______<br>________<br>________<br>SPECIFY:	

INSTRUMENT - FL_3:
Free Listing for Community Elders

SURVEY ID | **4-056**

Free Listing Exercises: ***Interviewer:*** *"Now I'm going to ask you questions about what people in your community think about certain issues. Let's get started. Please list as many things that come to your mind as you'd like."*

A. What are the biggest problems for pregnant women through delivery in the community?

B. What are the important tasks that elders in your community do regularly to care for pregnant women through delivery and the first few days after delivery?

C) Tell me what elders in your community know or believe about mothers' shelters?

D) What businesses or services are needed but not available in your community?

INTERVIEWER: "Thank you sincerely for your time. We have completed this interview and are grateful for your help as we work to develop or improve on access to skill deliveries in your community." RETURN TO THE COVER PAGE AND NOTE THE TIME THE INTERVIEW WAS COMPLETED.

ADDITIONAL NOTES OR COMMENT FROM THE INTERVIEWER:

INSTRUMENT - FL_3:
Free Listing for elders

SURVEY ID [ ]

Instrument ID: FL_3

Kuzumanana kuba abusena bwa kulindilila ba matumbu mucisi ca Zambia

FL Elders

Beleela:

1) Elders

Malailile kuli sikubuzya

Ntaamu 1: Cizuminano: *Amubuzye sikotola lubazu kwatuzuzumina tusyoonto ku ciindi cabo. Amulipandulule alimwi abumvwuntauzyi. Amutalike a cizuminano mbuli mbokuyisigwa. Na bazumina, amubape cipepa cacizuminano. Batola lubazu.*

Sena bazunina kutole lubazu kwiinda mukwambaula?

Iiyi ________ (zumanana amubandi)

Peepe ________ (amubalumbe basikutola lubazu kuciindi cabo pesi mutazumanani amubandi)

***Sikubuzya* :** *Bala kaambo katobela.. Twakomba amwiindiluke kaambo mumulaka ngoba mvwa ba mukabunga.*

"Twalumba kuti mwakazumina kutola lubazu mumumbandi ooyu. Izina lyangu ndime ______. Ndilamubuzya mibuzyo. Mweenzuma ______ unolemba zyomunoowaamba.

Tuyanda kuziba mbomuyeya kulanganya twaambo twamada akutumbuka kubusena nkomukkala. Kucita kuti maanda akulindilila bamatumbu azumanane, inga cayandika kuti bamatumbu nokuba bantu bamucooko kubbadela kusyonto kukkala mung'anda yabamatumbu kutegwa kaabeleka. Amulimwvwe kwangunuka kutwambila alimwi kufumbwa ncomuyanda kutwambila.

Alimwi mweleede kuziba kuti tamweleede kwaamba zintu zomutalimvwi kwanguluka kwaamba. Tatukalembi izina lyenu. Kunyina bwinguzi butali kabotu na bulikabotu, twakomba amusyomeke kutwambila camansimpe lwenu akuli basimukobonyoko, sena mwalibambila tutalike? "

Ntaamu 2: *Twalomba amutalike kubuzya mibuzyo yabukkale .*

Ntaamu 3: Amuzumanane, kwaambaula kwanguluka kulemba zitobela twalomba kuti mubuzisisye kujana bwini kutwaambo tunji tukatazya kumubuzyo amubuzyo. Na sikwingula kwina cimbincayeeya, zyakwaamba zyamana. Amupilukile kumalembe akutalika akubuzya makatazyo ajanika. Kuntaamu ya mamanino, amwiinduluke nzyomwali kukanana abupanduluzyi akuzyiba zyina lyamakatazyo nokuba bupanduluzyi. Kupenzi apenzi, amubuzye muntu ukonzya kumana penzi nokuba kuba aaluzyibo. Amulembe zyina lyakwe akkeyala yamuntu ooyo.

Ntaamu 4: Amuzumanane kumibuzyo yakulyaba kubbadela. Munobuzya sikuyingula mibuzyo kuziba na maanda akulindilila bamatumbu anobbadelwa kucita kuti azumanane.

Interviewer ID _________ **Note-taker ID:________________**

1. **Interview Date (DD/MM/YYYY)** ___________________
2. **Time Start** _______ **Time Finish** ___________
3. **Supervisor initials** __________

INSTRUMENT - FL_3:
Free Listing for elders **SURVEY ID**

Table 1: Respondent Demographics
Sikubuzya: *"ndilamubuzya mibuzyo imugama, yang'anda yenu ayamada enu a banabenu kasimbi/abazikulu benu madaa abakaintu beenu"*

Q#	QUESTION	CODE	Response	SKIP
000	Sikuingula / cibelesyo	Bakaintu FL_1: **(1)**<br>Balumi FL_2: **(2)**<br>Bapati FL_3: **(3)**	______________	
100.	Sikuingula mwalumi/mukaintu	Musankwa **(1)**<br>Musimbi **(2)**	______________	
101.	Mukkala kucooko cili?	Xxxxxx **(1)**<br>Xxxxxxx **(2)**<br>Xxxxxxx **(3)**<br>Xxxxxxx **(4)**	______________	
102.	Mwakali a myaka yongaye mwakali?	Myaka (___ ____)	______ _______	
103.	Sena mulikwetwe?	Kukwatwa/Kukalatomwe **(1)**<br>Kwanzana, kufwidwa kulekana **(2)**<br>Nabutema **(3)**	______________	
104.	Muli abana bongaye bapona?	Kunyina **000**<br>Nombolo ___		
104 A.	Ino mujisi bazikulu bongaye bapona?	Kunyina **000**<br>Nombolo ___		
105.	Kuli bana/bazikulu bongaye benu na bakulela bata siki myaka ili kumi alusele (18) bakkala mung'anda yenu ?	Kunyina **000**<br>Nombolo __	______________	
106.	Balibongaye bapati (kuvwela andinywe) balamyaka iinda ikkumi alusele (18) bakkala ang'anda yenu?	Kunyina **000**<br>Nombolo __		
107.	Sena mulabeleka?	Peepe **(0)**<br>Iiyi **(1)**		
108.	Maanda abamatumbu maanda akulindilila ba simada kabatanatumbuka kuti babe afwafwi azibbadela zijisi basiabupampu mukutumbusya ciindi cakutumbuka casika. Senakuli nomwakamvide maanda aya ?	Peepe **(0)**<br>Iiyi **(1)**	__________	
109.	**Mundambile mbomuzumina ku twaambo atu:**<br>Maanda abamatumbu alayandika kapati ku nseba zyabamatumbu abana bamvwanda	**(1)** Ndilazumina kapati<br>**(2)** Ndilazumina<br>**(3)** Ndilazumina/ tandizumini<br>**(4)** Ndilakaka<br>**(5)** Ndilakaka kapati	__________	
110.	Ncibotu kapati kumuntu mbuli muzukulu/mwana wangu musimbi/mukwe wangu kukkala mumaanda abamatumbu kwasyaala vwiki zisyoonto zya kutumbuka.		__________	
111.	Ndizinanzi lyacibbadela / kabbadela cili munisi-munsi ankomukkala?	Izina: _________<br>Tabazyi: 999	_______________	
[redacted]				
114.	Ino mwana wenu musimbi/muzikulu wenu wa	**(1)** Kunganda		

	tumbukila kuli calino-lino?	**(2)** Kukabbadela<br>**(3)** Kucibbadela<br>**(4)** Kumwi (kugame)	________________	
115.	Sena bana benu/ bazikulu benu balimitide?	Peepe **(0)**<br>Iiyi **(1)**<br>**Sezyi (99)**	___________	**Na Peepe koya ku 117**
116.	Sunu, muyeyakuti mwana wenu/ muzikulu wenu uuyotumbukila kuli?	**(1)** Kunganda<br>**(2)** Kabbadela<br>**(3)** Kucibbadela<br>**(4)** Kumwi (kugame)	___________	
117.	Sena bana benu/ bazikulu benu kuli nobakambilwa kuti bali a "buyumu yumu" bupati kumada abo?	Peepe **(0)**<br>Iiyi **(1)**<br>Sezyi **(99)**	Na iiyi, mwakacita buti?<br>___________	
118.	Sena kuli nkomuzyi kuunga mwana wenu /muzikulu wenu wakutumbukila kuko kuli bazi kutumbusya kabotu?	Peepe **(0)**<br>Iiyi **(1)**	___________	**Na Peepe koya ku 122**
119.	Mbusena nzi bwakabbadela / cibbadela cilafwafwi kobakonzya kujana balaluzyibo mukutumbusya kabotu?	Izina___________<br>Sezyi **(99)**	________________	
120.	Beenda buti kuzwa kobakala kusika kukabbadela / cibbadela kobanga bajana lugwasyo kuzwa kuliba syaabupampu batumbusya?	(1) Amaulu<br>(2) Amota ya kubbadela<br>(3) Amota<br>(4) Achiinga<br>(5) Acikochi<br>(6) Cinwi (cigame)		
121.	Cibatolela ciindi ciilamfwu buti kuzwa kobakkala kusika kubusena bula fwafwi kubelesya ceenzyo comwaamba?	(1) Kutasika woola lyoomwe<br>(2) Kwinda woola lyoomwe<br>(3) Kwinda mawoola obilo		
122.	Muciyanda bazikulu bambi?	Peepe **(0)**<br>Iiyi **(1)**<br>Sezyi **(99)**	___________	
123.	Nabatumbukila kukabbadela / cibbadela muyanda kuti bakkale mazuba ongaye bamana kutumbuka?	Nombolo____	_________	
124.	Kuti bayanda kukkala munganda yabamatumbu zyintu nzi nzyomuyanda kuti ba citilwe bamana kutu mbuka?<br><br>*Sala zyoonse zyeelede.*	**(1)** Kulangwalangwa asiku tumbusya mwamana kutumbuka<br>**(2)** Kuzumanana kunwa ma pilusi avitameni ada.<br>**(3)** Kulangwa kwa mwana mumvwanda kwainda mazuba ali osamwe akamwi akuyiisigwa<br>**(4)** Kuyiya kubamba mwana mumvwanda<br>**(5)** Kutantanya bana (family planning)<br>**(6)** Zimwi (zigame)	_________<br>_________<br>_________<br>_________	

INSTRUMENT - FL_3:
Free Listing for elders

SURVEY ID

Free Listing Exercises: ***Sikubuzya:*** *"eno ndiyanda kumubuzya mibuzyo mbuli mbo bayeeya bantu kucooko cenu munzintu zimwi. Atutalike. Twalomba mwaambe zintu zyoonse zyili kumoyokwenu.*

A. Mapenzi nzi mapati kuli bamakaintu bamada kucooko cenu?

Table 2: Mapenzi	Musyobo wamapenzi	Nguniwendelezya na ulaluzibo kumapenzi aya ? (izina cacita, nambala ya foni akubusena nkwakkala)
1.		
2.		
3.		
4.		
5.		
6.		
7.		
8.		
9.		
10.		

C) Mundambile eco bantu mucooko cenu cobazyi na cobasyoma kujatikizya maanda aba matumbu? (buzisisye kujana bwini bwa milawo na tunsia-nsia aluymwi zitobelwa, busena akwiindana kwa luzibo akati ka baalumi abamaintu alimwi a bapati / (bamacembele)

Table 4. Luzibo na lusyomo	Bupanduluzi	Nguniwendelezya na ulaluzibo kumapenzi aya ? (izina cacita, nambala ya foni akubusena nkwakkala)
1.		
2.		
3.		
4.		
5.		
6.		
7.		
8.		

9.		
10.		

D) Makwebonzi na ncito ziyandikana zitako kucooko cenu?

Table 5. Makwebo Na Ncito	Musyobo Wamakwebo	Nguniwendelezya na ulaluzibo kumapenzi aya ? (izina cacita, nambala ya foni akubusena nkwakkala)
1.		
2.		
3.		
4.		
5.		
6.		
7.		
8.		
9.		

Sikubuzya: "Ndalumba kapati ku ciindi cenu. Twamana mubandi oyu alimwi tuli lumbide kugwasya kwenu mbotuyobeleka kubamba na ku ba mbulula maanda abamatumbu mu cooko cenu." PILUKA KU PEPA LYA KUSANGUNA A KULEMBA CIINDI MUBANDI NOWAMANA.
