# Supplementary material for: Listening to the community: Using formative research to strengthen maternity waiting homes in Zambia
Source: PLoS One. 2018 Mar 15;13(3):e0194535. doi: 10.1371/journal.pone.0194535 (PMC5854412; doi:10.1371/journal.pone.0194535)
Supplement: S2 File — This is the study instrument used for the focus group discussions with women, men, TBAs/SMAGs, and mothers-in-law, in English and the local language of Tonga. (PDF) [file pone.0194535.s003.pdf]

INSTRUMENT - FG_1:
Focus Group Guide

GROUP ID | |

Instrument ID: FG_1

Sustainable Access for Waiting Mothers in Zambia

Focus Group Discussion Guide

Target Audiences:

1) Recently Delivered/Pregnant Women

2) Men with a child under 2 years

3) TBAs/SMAGs

4) Mothers-in-law

Instructions for the Interviewer

Step 1: Informed Consent: *Ask each participant for a few minutes of their time. Introduce yourself and the study. Begin the informed consent as per the training. If consent is granted, leave the informed consent sheet with each participant.*

Was verbal informed consent obtained for each participant before the start of the FGD?

YES ________ (proceed with FGD)

NO ________ (STOP! Thank the participants for their time but ask any who did not agree to leave. Then proceed with the FGD)

***Interviewer**: Read the following statement. Please repeat the statement translated into the local language based on primary languages used by the group.*

"Thank you for agreeing to participate in this interview. My name is ______. I will be asking you the questions. My partner ______ will be taking notes on the things you have to say.

We want to understand your views on pregnancy and delivery in your community. To make these mothers' shelters sustainable, it might be necessary for mothers or other community members to pay something to stay at the shelter to keep them functioning. Please feel free to tell us whatever you are comfortable sharing. You should also remember that you do not have to share anything that you are not comfortable sharing. We will not write down your name. There are no right or wrong answers, so please be honest and tell us what is true for you and your community. Are you ready to begin?"

Step 2: *Please complete the basic demographic table for each participant as they sign in. This form will have an ID number for each participant. Make sure that the note taker has the correct ID numbers recorded on his/her notes prior to beginning and that participants have their correct "Letter Label" hanging around their neck.*

Step 3: *Proceed to the FGD and mapping exercises. Start by reiterating the importance of confidentiality within the group. As you ask the questions, please probe to obtain as many problems as possible for each question. .*

Facilitator ID _________ **Note-taker ID:________________**

1. **FGD Date (DD/MM/YYYY)** __________________
2. **Catchment Area #: ______________**
3. **Time Start** _______ **Time Finish** ___________

Supervisor initials __________

INSTRUMENT - FG_1:
Focus Group Guide

GROUP ID [ ]

Demographics: Pregnant/Recently Delivered Women

ID	Age	Gender<br>**1)** Male<br>**2)** Female	Marital status:<br>**1)** Married<br>**2)** Widowed, separated, divorced<br>**3)** Single, never married	How many children do you have?	# children in household	# adults in house-hold	It is very worthwhile for someone like me (my wife/my daughter) to stay in a mothers' shelter during the last weeks of a pregnancy**	For this pregnancy or the most recent, where did/will you deliver?<br>(1) In a home<br>(2) Health facility<br>(3) Hospital<br>(4) Other (specify)	About how many KM do you live from the nearest health facility?
A									
B									
C									
D									
E									
F									
G									
H									
I									
J									

*1=Strongly agree, 2 = Agree ,3 = Neither agree nor disagree, 4=Disagree, 5=Strongly disagree

GROUP ID

Theme 1. Delivery practices and location

1.1 Tell me about the customs and traditions in your community for caring for women who are pregnant, giving birth, and the few days after delivery.

1.2 Where do most pregnant women in your community deliver their babies?

1.3 In your opinion, why do pregnant women who deliver at home do so instead of using a health facility?

Probe for issues related to:

- Access (distance, transport, cost)
- Permission or support from family members
- Household responsibilities
- Perceptions of health facility and quality of care
- Perception of pregnant woman's health risk
- Myths or misconceptions about home or facility delivery

1.4 In your opinion, why do pregnant women who deliver at a facility decide to do so?

Probe for issues related to:

- Access (distance, transport, cost)
- Permission or support from family members
- Household responsibilities
- Perceptions of health facility and quality of care
- Perception of pregnant woman's health risk
- Myths or misconceptions about home or facility delivery

1.5 In your opinion, where is the **best place** for pregnant women in your community to give birth? Why?

- What are the advantages of delivering at this place (identified location)?
- What are the disadvantages of delivering at this place (identified location)?

Theme 2: Awareness/perceived need/opinions/current usage of Mothers' Shelters

2.1 In your opinion, how could pregnant women in your community benefit from using a mother's shelter? Who would use the shelter most? Please explain.

- Probe for:
  - Need based on health reasons
  - Need based on geography (distance, accessibility in rainy season)
  - Need based on socioeconomic factors
  - Need based on gravida
  - Previous experience with birth in the facility

2.2 In your opinion, what are the reasons pregnant women might use or not use a mother's shelter? (PROBE FOR: social, cultural, economic structural factors. Your questions should explore the following types of reasons:

Probe for:

- Social/cultural reasons
- Financial implications

- Perceptions of health and personal safety
- Permission/support from family
- Encouragement or discouragement from peers

Theme 3. Mothers' shelters support, acceptability/services/staff/structure

3.1 What kind of **supports** would pregnant women need to obtain before using the mother's shelter?

Probe for:

- Whose support/permission would be needed
- Help with childcare/housekeeping during the absence
- Help with transport
- Help with costs
- Person to accompany

3.2 Which persons should be allowed to stay with pregnant women in the mothers' shelter?

Probe for:

- Children, including number and age
- Other family members
- Traditional birth attendants
- Other support persons

3.3 What would be the essential things (elements) that would make a mothers' shelter good for pregnant women and their families?

Probe for:

- Aspects of the structure
- Amenities, furnishings, supplies
- Issues related to food/cooking
- Types of staff on hand to assist women
- Other factors

3.4 Why might a pregnant woman in your community who is high-risk choose **not** to stay in the mother's shelter before delivery?

- What things make it <u>hard</u> for a woman to use the mother's shelter?

Theme 4: Management/functioning/maintenance of existing mother's shelter what's happening currently?

4.1 Describe how the mother's shelter in your community works.

Probe for:

- Who built it? Who owns it now?
- Who is in charge on a day to day basis? (clinic staff, Health committee, other?) What tasks do they do to run the shelter? *If a committee:* How does it work? (*How often do they meet? Where?)*
- Is anyone compensated for running it? (In what way? *How much are they compensated?)*

4.2 What types of activities this (person/group) does?

- Can you tell me how you think the management of the shelter is working?
- How could the mother's shelters be better managed?

4.3 How do women, families, and community members contribute to maintenance/upkeep of mother's shelter?
- Can you describe some of the activities?

4.4 Who provides the resources (e.g. food, money, etc.) to keep the mother's shelter open?
- What resources does the community contribute to the running or operation of the mother's shelter in your area?

4.5 What has been the greatest challenge associated with keeping a mother's shelter open?
- What can be done to overcome these challenges?

Theme 5: Ideas for the Future of Mother's Shelters

5.1 What would you do to improve the support in your community for mother's shelters?
- Community: "What can the community do to support the mother's shelters?"
- Coordination: "How could health facilities better work with mother's shelters? "

Theme 7: Needs Assessment

The following are questions and probes for the RA to use to generate discussion with the women.

1. In general, tell me about the experience of a pregnant woman in your community. Are experiences different based on age, class, or other factors? If so, how?
   Probe for:
   - Physical health or healthcare
   - Nutrition
   - Education or vocational services
   - Economic wellbeing
2. Tell me about the people or places from where a woman gets support during her pregnancy.
   Probe for:
   - Physical health or healthcare
   - Nutrition
   - Education or vocational services
   - Economic wellbeing
3. We'd like to ask you businesses that would be useful in your community but that might not be currently available. What are ideas for businesses that would help the community? (After a list is made, please order them in a priority list with numbers – 1 being most important…).
   Probe for:
   - Existing businesses that could be expanded upon
   - Ideas for new businesses or services

INSTRUMENT - FG_1:
Focus Group Guide

GROUP ID

Instrument ID: FG_1

Sustainable Access for Waiting Mothers in Zambia

Focus Group Discussion Guide

Target Audiences:

1)Recently Delivered/Pregnant Women

2) Men with a child under 2 years

3) TBAs/SMAGs

4) Mothers-in-law

Malailile kuli sikubuzya

Ntaamu 1: Cizuminano: *Amubuzye sikotola lubazu kwatuzuzumina tusyoonto ku ciindi cabo. Amulipandulule alimwi abumvwuntauzyi. Amutalike a cizuminano mbuli mbokuyisigwa. Na bazumina, amubape cipepa cacizuminano. Batola lubazu.*

Sena bazunina kutole lubazu kwiinda mukwambaula?

Iiyi _________ (zumanana amubandi)

Peepe _________ (amubalumbe basikutola lubazu kuciindi cabo pesi mutazumanani amubandi)

Sikubuzya : *Bala kaambo katobela.. Twakomba amwiindiluke kaambo mumulaka ngoba mvwa ba mukabunga.*

“Twalumba kuti mwakazumina kutola lubazu mumumbandi ooyu. Izina lyangu ndime _______. Ndilamubuzya mibuzyo. Mweenzuma _______ unolemba zyomunoowaamba.

Mwakasalwa abantu bamucilawo cenu kuti ndinwe nomuli aluzyibo na lwiiyo lunji amakani ajatikizya kumita na mada a mbokuyandika kuzyala. Na kutumbuka kabotu. Tuyanda kuti tuzyibe twaambo tomujisi amakani aa mada a kutumbuka mucilawo cenu a bulangizyi kumanganda a bamamama a mbotweelende kuzyikwabilia kutolelela kuti na zyayandika.

Amliwi mweleede kuziba kuti tamweleede kwaamba zintu zomutalimvwi kwanguluka kwaamba. Tatukalembi izina lyenu. Kunyina bwinguzi butali kabotu na bulikabotu, twakomba amusyomeke kutwambila camansimpe lwenu akuli basimukobonyoko, sena mwalibambila tutalike? “

Step 2: *Please complete the basic demographic table for each participant as they sign in. This form will have an ID number for each participant. Make sure that the note taker has the correct ID numbers recorded on his/her notes prior to beginning and that participants have their correct “Letter Label” hanging around their neck.*

Step 3: *Proceed to the FGD and mapping exercises. Start by reiterating the importance of confidentiality within the group. As you ask the questions, please probe to obtain as many problems as possible for each question.*

.

Facilitator ID __________ **Note-taker ID:**_________________

1. **FGD Date (DD/MM/YYYY)** ____________________
2. **Catchment Area #:** _______________
3. **Time Start** ________ **Time Finish** ____________

Supervisor initials ___________

INSTRUMENT - FG_1:
Focus Group Guide

GROUP ID [ ]

Demographics: Pregnant/Recently Delivered Women

ID	Myaka	mwalumi/ mukaintu 1)Musankwa 2) Musimbi	Sena mulikwetwe?: (1)Kukwatwa/kukkala atomwe (2) Kufwidwa/ Kwanzana, kulekana (3)Nabutema	Muli abana bongaye bapona?	Kuli bana bongaye benu na bakulela bata siki myaka ili kumi alusele (18) bakkala mung'anda yenu	Balibongaye bapati (kuvwela andinywe) balamyaka iinda ikkumi alusele (18) bakkala ang'anda yenu	NCibotu kapati kumuntu mbuli ndime kukkala mumaanda abamatumbu kwacaala vwiki zisyoonto zya kutumbuka.**	Kudaa eeli naa kuku mita kwa linolino, nkokuli nkomwakatumbukila na nkomuya kutumbukila? (1) kunganda (2) kuli basinceba (3) kucibbadela (4) kumwi (amukwaambe)	Kulampa mamayile ongaye oko nkomukkala kuyakusika kucibbadela cilaafwafwi?
A		2							
B		2							
C		2							
D		2							
E		2							
F		2							
G		2							
H		2							
I		2							
J		2							

*1=Strongly agree, 2 = Agree ,3 = Neither agree nor disagree, 4=Disagree, 5=Strongly disagree

INSTRUMENT - FG_1:
Focus Group Guide

GROUP ID

Theme 1. Delivery practices and location

1.1 Mudaambile kujatikizya mbomucita a tunsiya- nsiya mucooko cenu kujatikizya bamakaintu balaamada, bamakaintu batumbuka alimwi kwainda mazuba masyoonto bamana kutumbuka.

1.2 Kubusena kuli bunji bwababamakaintu balaamada mucooko cenu nkobatumbukila?

1.3 Mukuyeya kwenu, nkaambo nzi bamakaintu balaamada batumbukila ku nganda ncobacitila boobo kwiinda kwlnka kuka bbadela/cibbadela?

Buzisisya kuzintu zijatikizya:

- Kukziba (misizo, zyendelo, muubo)
- Kuzumizigwa na lugwasyo kuzwa kulibamukwasyi.
- Kulanganya milimo ya mung'anda.
- Mbuli mbobayeeyela kobasilikislwa abubotu bwaku bambwa
- Mboba yeeyela buyumu-yumu bunga bwajanika ku mukaintu ulada
- Tunsiya-nsiya azyambwa zitayelende zyakutumbukila kung'anda na kucibbadela

1.4 Mukuyeeya kwenu, nkaambo nzi bamakaimu balaamada batumbukila kili ba sinceba ncobacitila boobo?

Kapati kumakani ajatikizya:

- Kukziba (misizo, zyendelo, muubo)
- Kuzumizigwa na lugwasyo kuzwa kulibamukwasyi.
- Kulanganya milimo ya mung'anda.
- Mbuli mbobayeeyela kobasilikislwa abubotu bwaku bambwa
- Mboba yeeyela buyumu-yumu bunga bwajanika ku mukaintu ulada
- Tunsiya-nsiya azyambwa zitayelende zyakutumbukila kung'anda na kucibbadela

1.5 Mukuyeeya kwenu, kubusena kuli kubotu kwa bamakaintu balaamada mucooko cenu nkobeelende kutumbukila? Nkaambo nzi?

- Mbubotu nzi bujanika kutumbukila kubusena oobu (bwaambwa wawa)?
- Mbubi nzi bujanika kuli bamakaintu kutumbukila kubusena oobu (bwaambwa wawa)?

Theme 2: Awareness/perceived need/opinions/current usage of Mothers' Shelters

2.1 Mukuyeeya kwenu, mbubuti mbokunga bamakaintu balaamada mucooko cenu bajana bubotu kwiinda mukubelesya maanda abamatumbu? Mbanibayo belesya nganda eeyi kwiinda? Pandulula.

Buzisisya:

- Zintu ziyandika zijatikizya nseba
- Zintu ziyandika zijatikizya busena
- Zintu ziyandika zijatikizya buvubi.
- Zintu ziyandika kujatikizya nombolo yamada ngomwajisi.
- Luzibo luliko kale kutumbukila kubusena.

2.2 Mukuyeeya kwenu, Twaambo nzi basimada ncobakozya kwiibelesya na kutabelesya nganda yabamatumbu?:

Buzisisya:

- Bube/zilegwa
- Kubula mali/nakudula
- Mbobayeeyela bube bwanseba abukwabilizi.

- Kuzumizigwa lugwasyo kuzwa kuli bamukwasyi
- Kuyumikizigwa na kutyompokezegwa kuzwa kubeenzinyina
- Bubotu, amilimo iliko

Theme 3. Nganda ya bamamama mboyigwasya kwizumina, milimo babelesi, abubambe.

3.1 Kugwasilizya kuli buti bamakaintu balamada nkobayanda kujana kabatana kubelesya nganda yabamatumbu?

Buzisyisya:

- Nguni sikugwasya na kuzumizigwa kuyooyandika
- Ugwasya kulananya bana naa kubamba nganda ciindi nobatako
- Ugwasya cakwendela
- Ugwasya ca kubbadela
- Muntu uti musindikile

3.2 Mbabali bantu bazumizigwa kuyokkala abasimada kunganda yabamakaintu?

Buzisyisya:

- Bana, kubikkilizya mwelwe alimwi a myaka yabo
- Bamiwibantu bamukwasyi
- Basiku tumbusya bamu minzi
- Bambi bantu basikugwasilizya

3.3 Zintu nzi zikozya kugwasilizya kubamba nganda ya bamakaintu kuti ibote kuli basimada alimwi a mikwasyi yabo?

Buzisyisya:

- Bube bwanganda mboiyakidwe
- Zintu zyakubelesya, zyuno, zimwi zyaku belesya lyoonse.
- Zintu zijatikizya zyakulya akujika na kwakujikila
- Bantu bali buti bajanika baku gwasyilizya bamakaintu.
- Zimwi zintu

3.4 Ino nkaambo nziikuti mukaintu ulaada mucooko cenu ujisi buyumuyumu pati ncasala kutakkala ku nganda yaba makaintu katana tumbuka?

- Zintu nzi zipa buyumu-yumu kumukaintu kuti abelesye nganda yaba makaintu?
- Zintu nzi zyuba-uba kumukaintu zipa kuti abelesye nganda yaba makaintu?

Theme 4: Management/functioning/maintenance of existing mother's shelter what's happening currently?

4.1 Nguni ulanganya bwendelezyi bwa buzuba-abuzuba bwa nganda yabamatumbu mucooko cenu?

4.2 Milimo nzi (muntu na tubunga) njobabeleka?

- Kondambila mboyeeya kuti mbobabeleka?

4.3 Mbubuti bamakaintu, mikwasyi, alimwi abantu bamucooko mbobagwasilizya kubamba na kusalazya nganda yabamatumbu?

- Kopandulula imwi milimo icitika?

4.4 Nguni ubapa zyakubelesya (mbulicakulya, mali, azimwi) kutegwa nganda yabamatumbu kayijulidwe?
- Zintu nzi bantu bamucooko zyobapa kukubeleka na kubelesya nganda yabamatumbu kubusena bwenu?

4.5 Cintu nzi cipati cikatazya kubamba nganda yabamatumbu kuti kayijulidwe?
- Cinzi cikozya kucitwa kutegwa tuzunde penzi na mapenzi aaya?

Theme 5: Ideas for the Future of Mother's Shelters

5.1 Cinzi ncomunga mwacita kusumbula lugwasyo mucooko cenu kunganda yabamatumbu?
- Cooko: "Cinzi bamucooko cobakozya kucita kugwasilizya nganda yabamatumbu?"
- Bendelezyi: "Mbubuti mboinga nganda yabamatumbu yaendelezegwa?
- Kubelekela antomwe: "Mbubuti mbobanga bacibbadela babeleka kabotu aba nganda yabamatumbu? " .

Theme 7: Needs Assessment

1. Mucimo nzi bamakaintu balaamada mucooko cenu mbobabede muzibela eezyi: Sena kuli kwindana kuliko ku myaka, bube, na zimwi zintu? Na mboobo, mbubuti?

    - Bube bwamubili na kubamba mubili
    - Bulotwe
    - Lwiiyo na milimo njobabeleka
    - Buhubi mbobabede

2. Nkukuli basimada mucooko cenu nkobakozya kuunka kuti na bayanda lugwasyo? Sena kuli kugwasilizya kuliko mumasena aaya:

    - Bube bwamubili na inseba
    - Bulotwe
    - Lwiiyo na milimo njobabeleka
    - Buhubi mbobobede
    -

3. Tuyanda kumubuzya makwebo akozya kumugwasya kucooko cenu ayo atako cino ciindi.
- Mizeezo nzi yamakwebo na milimo eyo ikozya kugwasya kucooko.?
    - **Buzisisya:** Cobabelesya kujika, pobajajila mafoni, azimwi kutegwa bayeeye.
- Sena mulijisi makwebo na milimo yalo iliko kale jomuyanda kuti yiyumizigwe?
