# Supplementary material for: Listening to the community: Using formative research to strengthen maternity waiting homes in Zambia
Source: PLoS One. 2018 Mar 15;13(3):e0194535. doi: 10.1371/journal.pone.0194535 (PMC5854412; doi:10.1371/journal.pone.0194535)
Supplement: S3 File — This is the study instrument used for the key informant interviews, in English and the local language of Tonga. (PDF) [file pone.0194535.s004.pdf]

INSTRUMENT – KII_1:
Key Informant

SURVEY ID

Instrument ID: KII_1

Sustainable Access for Waiting Mothers in Zambia

Key Informant

Target Audiences: Key Informants selection will be finalized by Community Responses to Free Listing Exercises
1)Health Facility Staff
2)Distric Health Staff
3)Provincial Health Staff
4) Community Leaders

Instructions for the Interviewer

Step 1: Informed Consent: *Ask the participant for a few minutes of their time. Introduce yourself and the study. Begin the informed consent as per the training. If consent is granted, leave the informed consent sheet with the participant.*

Was verbal informed consent obtained?

YES ________ (proceed with interview)

NO ________ (STOP! Thank the participant for their time but do not proceed with the interview)

***Interviewer**: Read the following statement. Please repeat the statement translated into the local language based on primary languages.*

"Thank you for agreeing to participate in this interview. My name is ______. I will be asking you the questions. My partner ______ will be taking notes on the things you have to say.

You have been identified by people in your community as knowledgeable about some of the issues surrounding pregnancy and access to safe delivery. We want to understand your views on pregnancy and delivery in your community and your perspectives on mothers' shelters and how to sustain them if they are needed. Please feel free to tell us whatever you are comfortable sharing. You should also remember that you do not have to share anything that you are not comfortable sharing.. There are no right or wrong answers, so please be honest and help us to understand what is true for you and your community. Are you ready to begin?"

Step 2: *Please begin the interview with the demographic questions.*

Step 3: *Proceed to the Semi-structured interview guide. Please probe to obtain as in-depth and specific information you can.*

Step 4: *Proceed to the 'Willingness to Pay' questions. You will be asking respondents a set of questions to determine how much the mothers' shelter should charge and how to make them sustainable.*

Interviewer ID _________ **Note-taker ID:________________**

1. **Interview Date (DD/MM/YYYY)** ___________________
2. **Time Start** _______ **Time Finish** ___________
3. **Supervisor initials** __________

INSTRUMENT – KII_1:
Key Informant

SURVEY ID

Table 1: Respondent Demographics
Interviewer: *"I'm going to start by asking you questions about yourself, your household and your (or your wife's) pregnancies."*

Q#	QUESTION	CODE	Response	SKIP
000.	Respondent/Instrument type	KII_1: **(4)**	4 ______________	
100.	Respondent gender	Male **(1)**<br>Female **(2)**	______________	
101.	What catchment area do you live in?	Naluja **(1)**<br>Kanchele **(2)**<br>Batoka **(3)**<br>Mapanza **(4)**	______________	
101. A	What is your role in the community?	**1)**Health Facility Staff<br>**2)**Distric Health Staff<br>**3)**Provincial Health Staff<br>**4)** Headman<br>**5)** Chief<br>**6)** Pastor<br>**7)** Other: (specify) __________	____________<br>Affiliated entity:	
102.	How old were you at your last birthday?	Years (___ ____)	______ _______	
103.	What is your marital status?	Married/in union **(1)**<br>Widowed,separated,divorced **(2)**<br>Single, never married **(3)**	______________	
[redacted]				
105.	How many children, yours or others, under 18 live in your household?	None **000**<br>Number __	______________	
106.	How many adults (including yourself) over the age of 18 live in your household?	None **000**<br>Number __		
[redacted]				
108.	Mothers' shelters are homes where a pregnant woman can stay before she delivers, in order to be close to a facility which offers skilled obstetric care. Had you ever heard of Mothers' shelters before now?	No **(0)**<br>Yes **(1)**	___________	
109.	**Tell me how much you agree with statements:** Mothers' shelters are very important for the health of mothers and newborns	**(1)** Strongly agree<br>**(2)** Agree<br>**(3)** Neither agree nor disagree<br>**(4)** Disagree<br>**(5)** Strongly disagree	___________	
110.	It is very worthwhile for someone like myself or women in my community to stay in a Mothers' shelter during the last weeks of a pregnancy.		___________	
111.	What is the name of the nearest health facility to where you live?	Name: _________<br>Unknown: 999	_______________	

Semi Structured Interview Guide: ***Interviewer:*** *"Now I'm going to ask you questions to help me understand your community. Let's get started."*

Theme 1: Services for Pregnant Women

<u>INSTRUMENT – KII_1:</u>
Key Informant

SURVEY ID

1.1 Describe the situation of pregnant women in *your community*? Are there some pregnant women in your community that are suffering more or are more vulnerable? Tell me about them and why they are more vulnerable. What are the greatest needs for pregnant women in your communities?

1.2 Please describe any services specifically for highly vulnerable women and families *in your community*?

Probe for:

- Nutrition
- Health care access/ Availability of clean delivery kits/

1.3. What is going well in providing services to pregnant women? What services are not going well and need to be improved? Please explain. Who should take responsibility for improvements and how should they do it? Specifically ask about:

- availability of CDKs
- Clinic ability to manage complications
- How to improve access for those far away? What is the best way to improve access?

Theme 2. Delivery practices and location

2.1 Tell me about the customs and traditions in your community for caring for women who are pregnant, giving birth, and the few days after delivery.

2.2 Where do most pregnant women in your community deliver their babies? **Why do they do so?** Probe: home, health center, hospital, en route to the clinic…

2.3 Community members have suggested several reasons why pregnant women who deliver at home do so. I'll read them to you. Please help me to better understand the issue from your perspective, whether you agree with the issue or not.

Issues:

- Access problems (distance, transport, cost)
- Difficulty meeting requirements of health facility for delivery: Women must bring Clean Delivery Kit, Baby clothes, food, other things to the clinic
- Limited skilled staff at the health facility.
- Poor facilities (e.g. no light for delivery, other delays in care).

2.4 In your opinion, why do pregnant women who deliver at a facility do so?

Theme 3: Awareness/perceived need/opinions/current usage of Mother's Shelters

3.1 In your opinion, how, if at all, could pregnant women in your community benefit from using a mother's shelter? Who would benefit most (who is most vulnerable)? Why?

Theme 4: Management/functioning/maintenance of existing mother's shelter: what's happening currently?

4.1 Describe how the mother's shelter in your community works.

Probe for:

- Who built it? Who owns it now?
- Who is in charge on a day to day basis? (clinic staff, Health committee, other?) What tasks do they do to run the shelter?How does it work? (*How often do they meet? Where?)*

4.2 How do women, families, and community members currently contribute to maintenance/upkeep of mother's shelter?

- Can you describe some of the activities? (drawing water, slashing, sweeping, weeding, cash, etc.)

4.3 Who provides the resources (e.g. food, money, etc.) to keep the mother's shelter open?

- What resources does the community currently contribute to the running or operation of the mother's shelter in your area? What or how could the community contribute more?

4.4 What has been the greatest challenge associated with keeping a mother's shelter open and operating?

- Probe for: additional patients/others staying, safety, lack of facilities
- What can be done to overcome these challenges?

Theme 5: Ideas for the Future of Mother's Shelters

5.1 What would you do to improve the support in your community for mother's shelters?

- Community: "What can the community do to better support the mother's shelters?"
  - o We've heard that there's a willingness for mothers to do small work when they stay at the shelter. What type of work/contribution would you suggest
  - o Community elders and other community members have suggested they'd pool some maize, chickens or money to contribute to the shelter. How do you think this could work?

5.2 Describe how you think a mother shelter should best be run. Please take your time to think about the best way we could make a shelter run well and be sustainable.

Probe for:

- Who should be in charge or involved?
  - a. Individual
  - b. Small business owner
  - c. Safe motherhood groups
  - d. Health facility staff
  - e. Pastor/church
  - f. Chief/headman?
- How should it work? (What activities should they do? If committee, when should they meet? How often? Who should be on the committee? How are they chosen?)
- Should people be compensated for running it? What are the advantages and disadvantages of trying to compensate or reward people for running the shelter?
-

5.3 Describe what services should be available for women who choose to stay at a mothers' shelter:

Theme 6: Coordinated Care Systems and Referral to Mother's Shelters

6.1 I'm going to read to you some challenges to accessing or using mothers' shelters for increasing access to facility deliveries that members of your community have identified. For each one, please help us

think of ideas to resolve some of the challenges, even if you don't agree with the challenge. Please be as specific as possible:

- Distance/difficult to access transport and no ambulance available:
- Women choose not to come because they don't have resources needed (e.g. CDK,baby clothes, food etc.):
- Women feel unsafe because anyone stays there including families of other patients

Theme 7: Community Needs

7.1. We are trying to identify ways that mother's shelters could be self-sustaining. We've asked about contributions of individuals and the community already. We are also thinking that a successful business opportunity could help to sustain the shelters and contribute financially. Community members have suggested several services and businesses that would be useful in their communities but are not currently available. I'll read you a list of ideas and you tell me how these ideas could generate income, and who you think would be helpful for me to contact to learn more about this: (*interviewer: probe with how exactly these ideas could generate income – charging for services, etc)*

- If the shelter were built with an extra room for an extra business, what do you think people in your community would like to be sold? (Is there another business that could use this space? How?
- Do you have other ideas that would help the community and contribute to mothers' shelters?

7.2. We are interested in learning how to best sustain mothers' shelters. Think about business or services (existing or not). Even if they are not related to health at all, do you have ideas about how businesses could contribute to the functioning of mother's shelters?

- Describe how you think this could work?
- **Probe with:** Profit contributions, public private partnerships, donations, in-kind assistance, shared business, etc.

7.3. Please think as broadly and creatively as you can. Do you have other ideas of how mothers' shelters could be managed and maintained by your community? Please describe.

INTERVIEWER: "Thank you sincerely for your time. We have completed this interview and are grateful for your help as we work to develop or improve on the mothers' shelters in your community. If in the next couple of days you think of an idea to better run or maintain a shelter or solve an access issue we discussed, please beep " RETURN TO THE COVER PAGE AND NOTE THE TIME THE INTERVIEW WAS COMPLETED.

INSTRUMENT – KII_1:
Key Informant

SURVEY ID [ ]

Instrument ID: KII_1

Sustainable Access for Waiting Mothers in Zambia

Key Informant

Target Audiences: Key Informants selection will be finalized by Community Responses to Free Listing Exercises
1)Health Facility Staff
2)District Health Staff
3)Provincial Health Staff
4)Community Leaders

Malailile kuli sikubuzya

Ntaamu 1: Cizuminano: *Amubuzye sikotola lubazu kwatuzuzumina tusyoonto ku ciindi cabo. Amulipandulule alimwi abumvwuntauzyi. Amutalike a cizuminano mbuli mbokuyisigwa. Na bazumina, amubape cipepa cacizuminano. Batola lubazu.*

Sena bazunina kutole lubazu kwiinda mukwambaula?

Iiyi ________ (zumanana amubandi)

Peepe ________ (amubalumbe basikutola lubazu kuciindi cabo pesi mutazumanani amubandi)

Sikubuzya : *Bala kaambo katobela.. Twakomba amwiindiluke kaambo mumulaka ngoba mvwa ba mukabunga.*

"Twalumba kuti mwakazumina kutola lubazu mumumbandi ooyu. Izina lyangu ndime ______. Ndilamubuzya mibuzyo. Mweenzuma ______ unolemba zyomunoowaamba.

Mwakasalwa abantu bamucooko cenu kuti ndinywe nomuli aluzyibo na lwiiyo lunji amakani ajatikizya kumita na mada. Na kutumbuka kabotu. Tuyanda kuti tuzyibe twaambo tomujisi amakani aa mada a kutumbuka mucooko cenu a bulangizyi kumaanda a bamatumbu tumbu kutiazwidilele kubeleka na ala yandika. Twalomba amwangunuke kutwambila zyomuyanda kwamba.

Amliwi mweleede kuziba kuti tamweleede kwaamba zintu zomutalimvwi kwanguluka kwaamba. Tatukalembi izina lyenu. Kunyina bwinguzi butali kabotu na bulikabotu, twakomba amusyomeke kutwambila camansimpe lwenu akuli basimukobonyoko, sena mwalibambila kutalike? "

Ntaamu 2: *Twalomba amutalike kubuzya mibuzyo yabukkale .*

Ntaamu 3: Amuzymanane kutobela mubandi. Twalomba mubuzisisye kujana bwini buzwide.

Ntaamu 4: Amuzumanane kumibuzyo yakulyaba kubbadela. Munobuzya sikuyingula mibuzyo kuziba na maanda akulindilila bamatumbu anobbadelwa kucita kuti azumanane.

Interviewer ID _________ **Note-taker ID:________________**

1. **Interview Date (DD/MM/YYYY)** __________________
2. **Time Start** _______ **Time Finish** ___________
3. **Supervisor initials** __________

INSTRUMENT – KII_1:
Key Informant and WTP

SURVEY ID

Table 1: Table 1: Respondent Demographics
Sikubuzya: *“ndilamubuzya mibuzyo imugama, yang’anda yenu ayamada enu.” (Amada abamakaintu benu)*

Q#	QUESTION	CODE	Response	SKIP
000.	Sikuingula / cibelesyo	KII_1: **(4)**	**4**<br>_____________	
100.	Sikuingula mwalumi/mukaintu	Musankwa **(1)**<br>Musimbi **(2)**	_____________	
101.	Mukkalal kucooko cili?	Naluja (**1**)<br>Kanchele (**2**)<br>Batoka (**3**)<br>Mapanza (**4**)	_____________	
101. A	Mujisi mulimo nzi mucooko eci?	**1**) musilisi wakabbadela<br>**2)** mubelesi wanseba acitilikiti<br>**3**) mubelesi wanseba kucooko ca ku musanza<br>**4)** sibbuku<br>**5)** Mwami<br>**6)** Mufwundisi<br>**7)** Zimwi: (Zingame) _________	_____________<br>Affiliated entity:	
102.	Mwakali a myaka yongaye mwakali?	Myaka (___ ____)	______<br>_______	
103.	Sena mulikwetwe?	Kukwatwa/kukkala atomwe (**1**)<br>Kufwidwa/Kwanzana, kulekana **(2)**<br>Nabutema **(3)**	_____________	
[redacted]				
105.	Kuli bana bongaye benu na bakulela bata siki myaka ili kumi alusele (18) bakala mung’anda yenu ?	Kunyina **000**<br>Nombolo __	_____________	
106.	Balibongaye bapati (kuvwela andinywe) balamyaka iinda ikkumi alusele (18) bakala ang’anda yenu?	Kunyina **000**<br>Nombolo __		
[redacted]				
108.	Maanda abamatumbu maanda akulindilila ba simada kabatanatumbuka kuti babe afwafwi azibbadela zijisi basiabupampu mukutumbusya ciindi cakutumbuka casika. Senakuli nomwakamvide maanda aya ?	Peepe (**0**)<br>Iiyi (**1**)	__________	

109.	**Mundambile mbomuzumina ku twaambo otu:**<br><br>Maanda abamatumbu alayandika kapati ku nseba zyabamatumbu abana bamvwanda	**(1)** Ndilazumina kapati<br>**(2)** Ndilazumina<br>**(3)** Ndilazumina/ natandizumini<br>**(4)** Ndilakaka<br>**(5)** Ndilakaka kapati	__________	
110.	NCibotu kapati kumuntu mbuli ndime aba bamakaintu kukkala mumaanda abamatumbu kwacaala vwiki zisyoonto zya kutumbuka.		__________	
111.	Ndizinanzi lyacibbadela / kabbadela cili munisi-munsi ankomukkala?	Izina: _________<br><br>Tabazyi: 999	____________<br>__	

Semi Structured Interview Guide: ***Interviewer:*** *"Eno ndiyomubuzya mibuzyo. Lindigwasya. Kuzibisisya cooko cenu atutailike."*

Theme 1: Services for Pregnant Women

1.1 Pandulula bube bwababamakaintu baliaamada mucooko cenu ? Sena nkobali bamakaintu balaamada mucooko cenu ba pengende a bapengende kapati? Kamundaambila cijatikizya mba'bo alimwi nkaambo nzi ncobapengede maningi? Zintu nzi zyipati-pati zyiyanda bamaikaintu balaamada muzyooko cenu?

Buzisisya:

- Bube bwanseba, kabatana tumbuka, bulotwe (zyakulya zikwene).
- Kuti zintu ziyandi kapati zyajanika, nganda alimwi a zyakulya.
- Bulamfwu bwamisinzo

1.2 Twalomba amupandulule imwi milimo kapati kuli bamakaintu bapengende a mikwasyi mucooko cenu?

Buzisisya:

- Bulotwe
- Lugwasyo kumakani anseba

1.3. Zintu nzi zyeenda kabotu mukugwasilizya bamakaintu balaamada? Zinzi zyiyandika kubambululwa? Kopandulula. Nguni weelende kubweza milimo yalusumpuko alimwi beelende kucita buti?

Theme 2. Delivery practices and location

2.1 Mudaambile kujatikizya mbomucita a tunsiya- nsiya mucooko cenu kujatikizya bamakaintu balaamada, bamakaintu batumbuka alimwi kwainda mazuba masyoonto bamana kutumbuka.

Buzisisya:

- Nguni kujanika lyoonse kuciindi cakumyongwa? Ino muntu ooyu ucita nzi kumukaintu na myongwa?
- Nguni ujanika kuciindi cakutumbuka? Ino muntu ooyu ucita nzi ciindi natumbuka mukaintu?

- Sena kuli muntu umbi ulanga mwana watumbukwa na mbabona balanga banyina? Ino nguni muntu ooyu (na mbaani bantu aba)?
- Zintu nzi zizibidwe zyomucita kuli banyina alimwi akumwana kufwambaana amana kutumbukwa? Amu nvwiki zitobela?
- Zintu nzi zizibidwe zyomucita kumwana amana biyo kutumbukwa?
- Ciindi cilamfwu buti mukaintu cayelende kukkala (kubusenabusalidwe) amana kutumbuka?

2.2 Kubusena kuli bunji bwababamakaintu balaamada mucooko cenu nkobatumbukila? Nkaambo nzi ncobasalila kucita boobo?

2.3 Mukuyeya kwenu, nkaambo nzi bamakaintu balaamada batumbukila ku nganda ncobacitila boobo kwiinda kwlnka kuka bbadela/cibbadela?

Buzisisya kuzintu zijatikizya:
- Kukziba (misizo, zyendelo, muubo)
- Kuzumizigwa na lugwasyo kuzwa kulibamukwasyi.
- Kulanganya milimo ya mung'anda.
- Mbuli mbobayeeyela kobasilikislwa abubotu bwaku bambwa
- Mboba yeeyela buyumu-yumu bunga bwajanika ku mukaintu ulada
- Tunsiya-nsiya azyambwa zitayelende zyakutumbukila kung'anda na kucibbadela

2.4 Mukuyeeya kwenu, nkaambo nzi bamakaimu balaamada batumbukila kili ba sinceba ncobacitila boobo?

Kapati kumakani ajatikizya:
- Kukziba (misizo, zyendelo, muubo)
- Kuzumizigwa na lugwasyo kuzwa kulibamukwasyi.
- Kulanganya milimo ya mung'anda.
- Mbuli mbobayeeyela kobasilikislwa abubotu bwaku bambwa
- Mboba yeeyela buyumu-yumu bunga bwajanika ku mukaintu ulada
- Tunsiya-nsiya azyambwa zitayelende zyakutumbukila kung'anda na kucibbadela

Theme 3: Awareness/perceived need/opinions/current usage of Mother's Shelters

3.1 Mukuyeeya kwenu, mbubuti mbokunga bamakaintu balaamada mucooko cenu bajana bubotu kwiinda mukubelesya maanda abamatumbu? Mbanibayo belesya nganda eeyi kwiinda? Pandulula.

Buzisisya:
- Zintu ziyandika zijatikizya nseba
- Zintu ziyandika zijatikizya busena
- Zintu ziyandika zijatikizya buvubi.
- Zintu ziyandika kujatikizya nombolo yamada ngomwajisi.
- Luzibo luliko kale kutumbukila kubusena.

Theme 4: Management/functioning/maintenance of existing mother's shelter: what's happening currently?

4.1 Pandulula mbuli bendelezyi ba nganda yabamatumbu mucooko cenu mbobabeleka buzuba-buzuba?

Buzisisya:

- Wendelezya?
- Cinzi muntu tubunga ncobacita?
- Ino cibeleka buti?
- Ino babbadelwa buti mukubeleka ooku?

4.2 Mbubuti bamakaintu, mikwasyi alimwi abantu bamu-cooko mbobagwasilizya kubamba nganda ya bamamatumbu?
- Kopandulula zimwi zintu zyobacita?

4.3 Nguni ugwasilizya na uupa zyintu (mbuli cakulya, mali, azimwi) kutegwa nganda yabamatumbu kaizumanana kubeleka?
- Zintu nzi basicooko zyobapa kukubeleka na kukubelesya kwa nganda yabamatumbu kubusena bwenu?

4.4 Zintu nzi zyalikupa buyumuyumu kapti kuti nganda yabamatumbu kaizumanana kubeleka?
- Cinzi cikozya kucitika kutegwa tuzude buyumu-yumu oobu?

Theme 5: Ideas for the Future of Mother's Shelters

5.1 Ncinzi ncomunga mwacita kuyungizya lugwasyo mucooko cenu ku nganda yabamatumbu?
- Basicooko: "Ino basicooko inga bacita nzi kutegwa bagwasilizye kabotu nganda yabamatumbu?"
- Beendelezyi "Ino mbubuti nganda yabamatumbu mboinga yeendelezyegwa?"
- Amubuzisisya zitondezyo zigaminina

5.2 Amu pandulula mbomuyeya kuti nganda yabamatumbu kaibelesegwa:
Buzisisya:
- Nguni weelede kwendelezya?
  - *a.* Muntu omwe
  - b. Bamakwebo masyoonto
  - c. Tubanga tulanganya kutumbuka kabotu
  - d. Babelesi bacibbadela
  - e. Mufwundisi cikombelo
- Ino ceelede kubeleka buti?
- Ino bantu bayelede kumbadelwa buti kwendelezya?
- Milimo nzi yeelede kucitwa?

5.3 Pandulula milimo iyeelede kuti kazijanika ya bamaka-intu basala kukkala ku nganda yabamatumbu:
Buzisisya:
- Kulabwa lugwa mada abasyaabupampu.
- Kujanika kwazibelesyo syakutumbusya
- Kulagwa bamana kutumbuka-kubamba mwana kabotu kunyosya kukanana, azimwi.
- Zyendelo

Theme 6: Coordinated Care Systems and Referral to Mother's Shelters
6.1 Pandulula mbomukozya kujana milimo na lugwasyolwa bamakaintu balaamada kutegwa babelekele atomwe mucooko eci?
- Sena nkuli nzila zikozya kusumpula milimo kuli bamakaintu balaamada alimwi ababo bakatumbuka eno-eno?
- Ino buyumuyumu nzi bwakubelekela atomwe alimwi alugwasyomucooko cenu?

- Ino inga mwacita nzi kusumpula milimo yakubelekela atomwe mucooko cenu kuti, na mwakozya?

Theme 7: Community Needs

7.1. Tuyanda kumubuzya makwebo akozya kumugwasya kucooko cenu ayo atako cino ciindi.

- Mizeezo nzi yamakwebo na milimo eyo ikozya kugwasya kucooko.?
    - **Buzisisya:** Cobabelesya kujika, pobajajila mafoni, azimwi kutegwa bayeeye.
- Sena mulijisi makwebo na milimo yalo iliko kale jomuyanda kuti yiyumizigwe?

7.2. Tulayandisisya kwiiya mbotukozya kucita kuti maanda a bamatumbu azwidililie kubeleka. Amuyeeye makwebo na milimo (iliko na itako). Nokuba kuti tazijatikizyi ku nseba, sena mulijisi mizeezo ya makwebo aya inga yacita kuti maanda akulindilila kabeleka?

- Amupandulule mbo muyeeya kuti inga abeleka?
- **Buzisisya:** Mpindu zipegwa, tubunga twabuleya tutali twamfwulumenda zyipego, kugwasigwa, makwebo akubelekela antomwe, zimwi.

7. 3 Twalomba muyeeye cabusongo mbuli mbomukozya. Sena mulijisi mizeezo imbi yakubamba akubambulula maanda abamatumbu mucooko cenu? Twalomba amupandulule.

Sikubuzya: "Ndalumba kapati ku ciindi cenu. Twamana mubandi oyu alimwi tuli lumbide kugwasya kwenu mbotuyobeleka kubamba na ku ba mbulula maanda abamatumbu mu cooko cenu." PILUKA KU PEPA LYA KUSANGUNA A KULEMBA CIINDI MUBANDI NOWAMANA.
